# Supplementary material for: Combined 1H-Detected Solid-State NMR Spectroscopy and Electron Cryotomography to Study Membrane Proteins across Resolutions in Native Environments
Source: Structure. 2018 Jan 2;26(1):161–170.e3. doi: 10.1016/j.str.2017.11.011 (PMC5758107; doi:10.1016/j.str.2017.11.011)
Supplement: Document S1. Figures S1–S4 [file mmc1.pdf]

Structure, Volume *26*

Supplemental Information

Combined ^{1}H-Detected Solid-State NMR Spectroscopy and Electron Cryotomography to Study Membrane Proteins across Resolutions in Native Environments

Lindsay A. Baker, Tessa Sinnige, Pascale Schellenberger, Jeanine de Keyzer, C. Alistair Siebert, Arnold J.M. Driessen, Marc Baldus, and Kay Grünewald

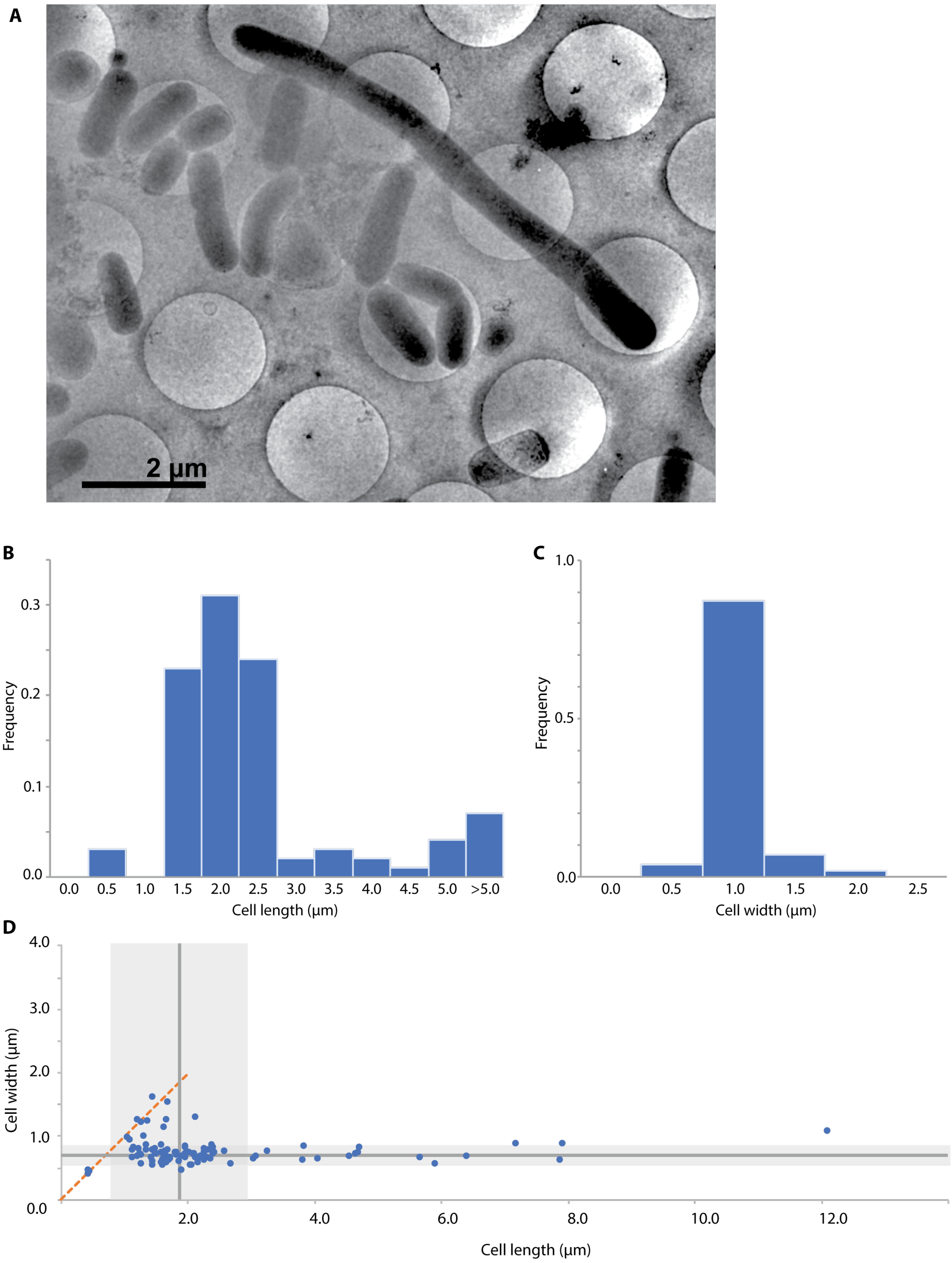

Supplemental Figure 1. (Related to Figure 2) The effect of rifampicin treatment on *E. coli* BL21 morphology. **A**) CryoEM projection image of *E. coli* BL21 expressing YidC after 15 hr rifampicin treatment at 25°C. Quantification of average cell length (**B**) and cell width (**C**) after rifampicin treatment. Most cells were between 1.0-2.5 µm long and 0.5-1.0 µm wide, but some were longer (<20%). **D**) Comparison of cell length versus cell width. Most cells maintained their rod shape, but a few exhibited a spherical morphology (orange dotted line). The median cell length was 1.86 µm (vertical grey line) with a standard deviation of the mean of 1.06 µm (shaded area either side). Typical cell lengths for *E. coli* when grown in glucose are 1.7-2.6 µm (Taheri-Araghi et al., 2015). The median cell width was 0.71 µm (horizontal grey line) with a standard deviation of the mean of 0.14 µm (shaded area either side).

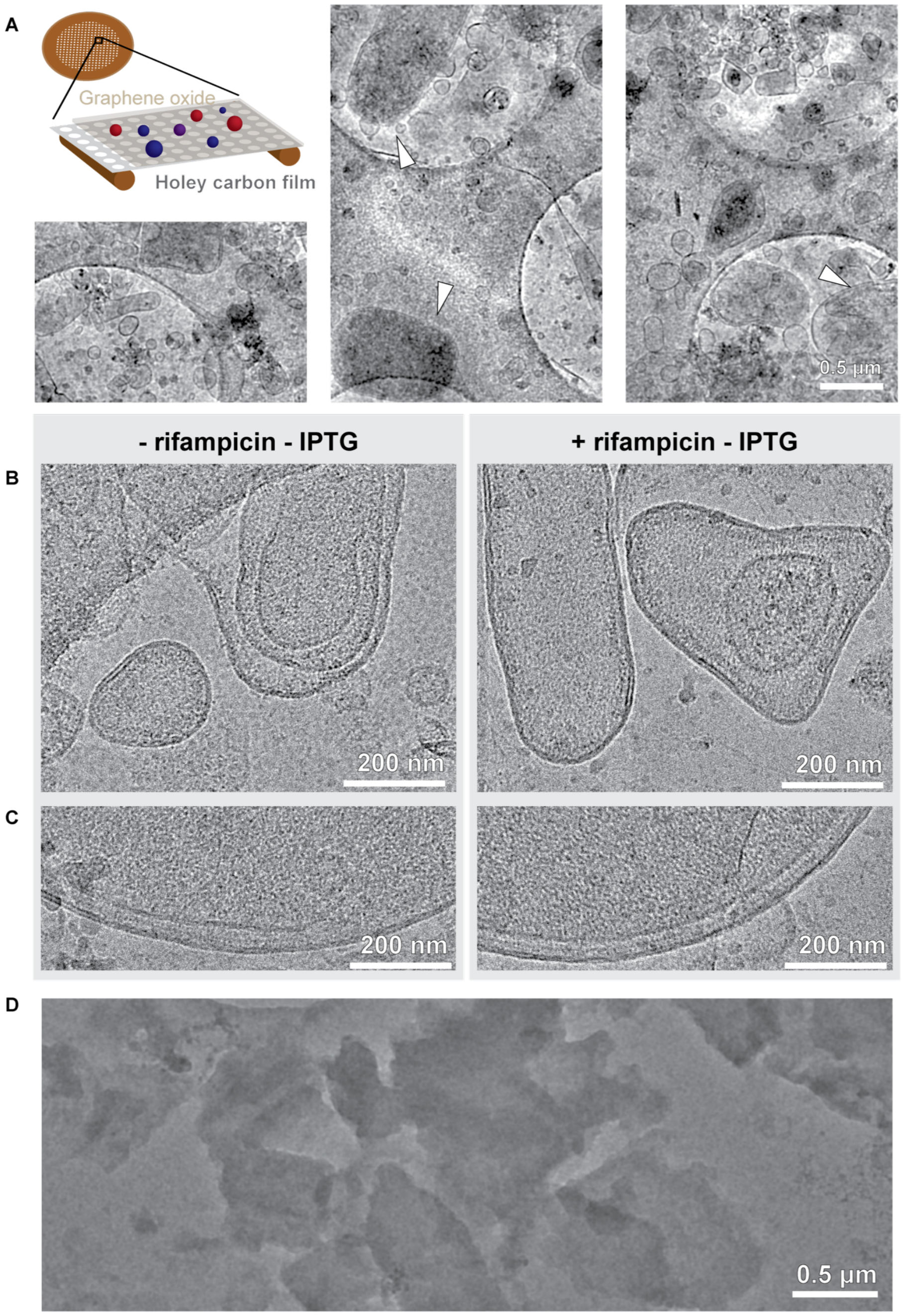

Supplemental Figure 2. (Related to Figure 2) Comparison of the morphology of cell envelope preparations with and without rifampicin and YidC over-expression. **A**) Cell envelope samples after rifampicin treatment and heterologous protein expression on graphene-oxide coated holey carbon grids (schematic, inset) display a variety of morphologies, including those that maintain the native cell pole (white arrows). **B**) No significant differences in the size or shapes of membranes was seen with or without rifampicin or YidC over-expression. **C**) No differences were observed in cell wall formation with or without rifampicin and YidC over-expression. **D**) Manipulation of cell envelope samples by standard methods used with synthetic bilayers (such as extrusion, freeze-thaws or sonication) produced a sheet morphology which is unsuitable for cryoET.

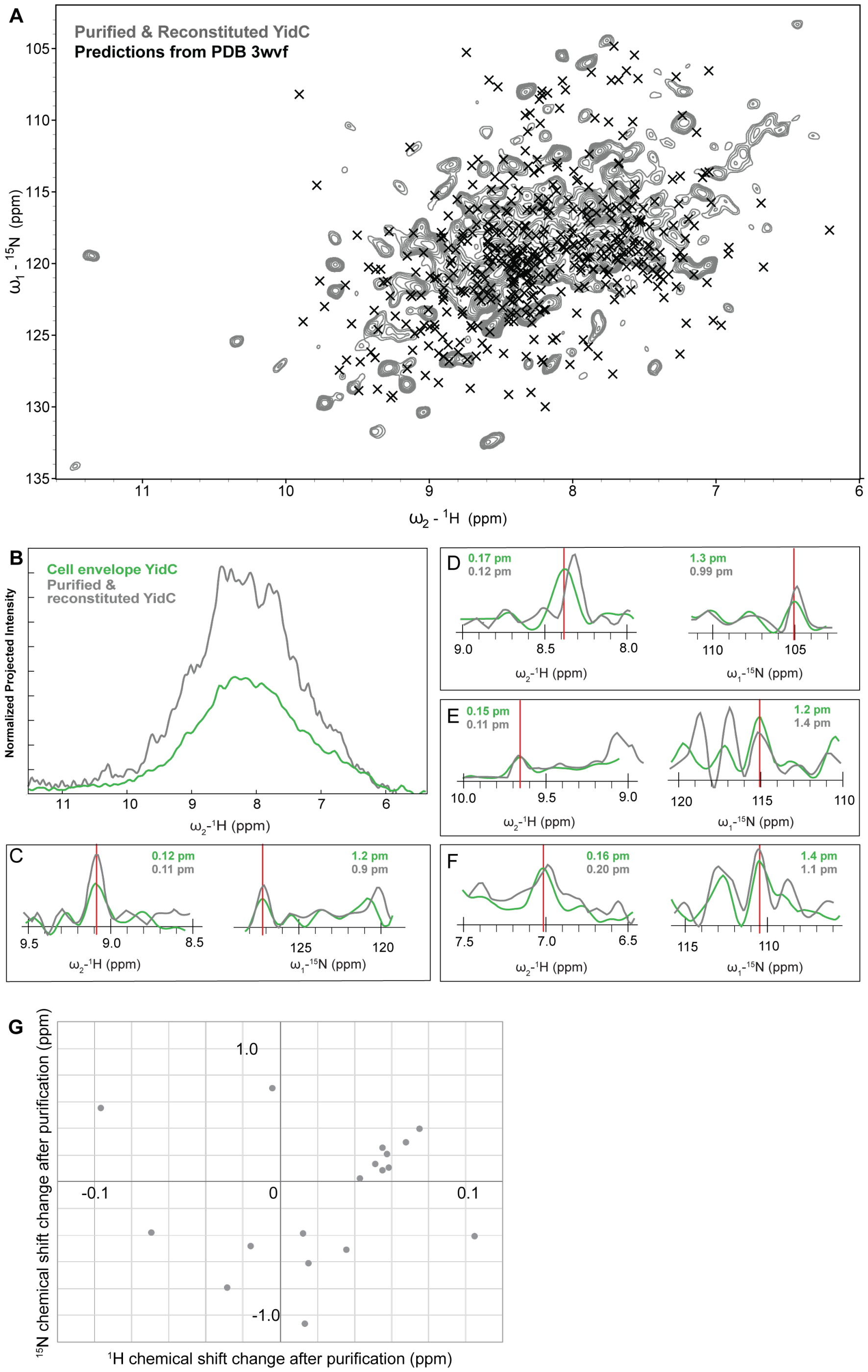

Supplementary Figure 3. (Related to Figure 3) **A)** Predictions of ssNMR resonances (black crosses) from Shiftx2 (Han et al., 2011) using the crystal structure of YidC (Kumazaki et al., 2014) (PDB 3wvf) do not agree well enough with our observed ssNMR spectrum (grey – purified and reconstituted YidC as in Figure 3) to assign resonances. **B)** Projection in the ^{15}N dimension of the 2D spectra from figure 3, normalized according to the square root of the number of scans used to acquire each spectrum. The concentration of YidC in the cell envelope samples is ~ 2x less than in the purified & reconstituted sample. **C-F)** Pairs of slices through the spectra in Figure 3 (cell envelope YidC – green; purified and reconstituted YidC – grey) for isolated peaks in the ^{1}H dimension (left) and ^{15}N dimension (right). The vertical red line in each spectrum indicates the position of the slice in the other dimension. The full width at half maximum intensity of the peak closest to red line is given in a top corner for both samples. Overall, line widths are often similar when comparing the two samples. **G)** Scatter plot showing the change in chemical shift for resolved peaks (assuming that the closest nearby correlation represents the same protein residue) in the purified and reconstituted spectrum in Figure 3, relative to the cell envelope spectrum.

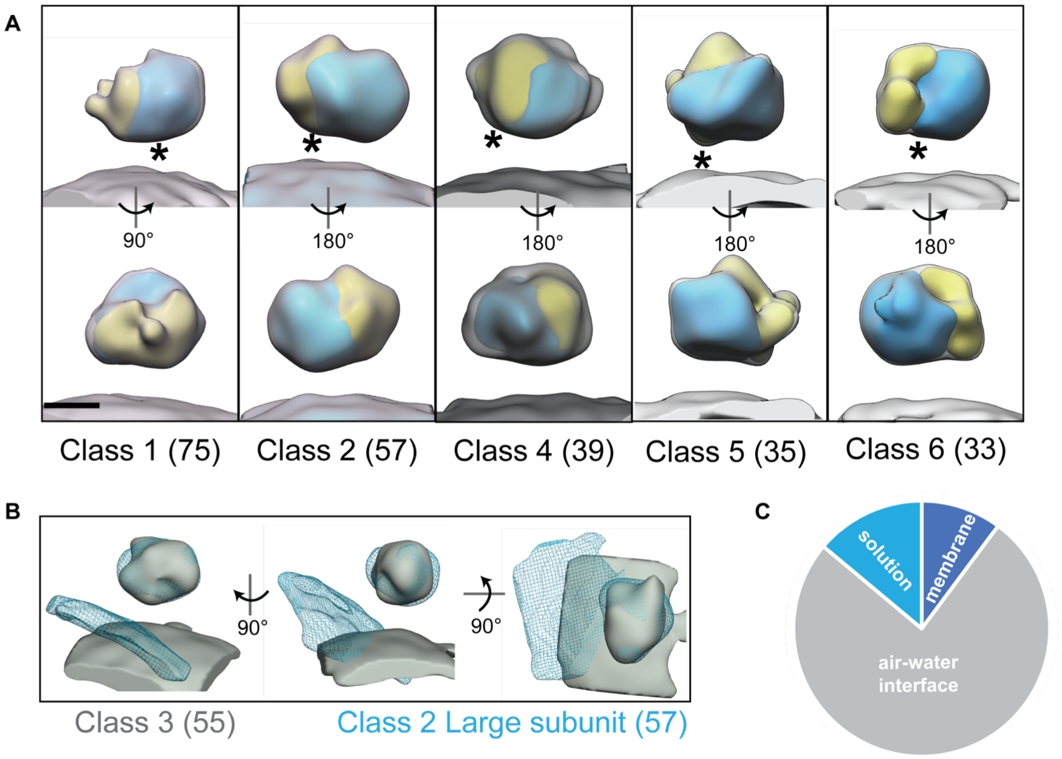

Supplementary Figure 4. (Related to Figure 5) Sub-tomogram averaging of YidC cell envelope-associated RNCs with Peet (Heumann et al., 2011). After aligning to an membrane-oriented average of all and then classifying with principle component analysis (PCA), particles were aligned to the class with the RNC in a YidC binding-competent conformation and then further classified by PCA. **A**) Five of the six classes (grey) with sufficient numbers for averaging were in YidC binding-competent positions, as judged by orientations of the large (blue) and small (yellow) ribosomal subunits (segmented manually) relative to the membrane. The approximate location where nascent chain would emerge from the exit channel is marked with an asterisk (*). Scale bar 10 nm. **B**) One class (grey) only had density for the large ribosomal subunit, which was in a different orientation relative to the membrane than those in the other classes (class 2 large subunit and membrane shown as blue mesh). **C**) Only ~25% of observed RNCs were available for analysis, due to the large number interacting with the air-water interface. As the system had time to reach equilibrium, this air-water interaction reduces the total concentration of RNCs available for binding but not the relative ratio of bound/unbound.
