# Supplementary material for: Combined 1H-Detected Solid-State NMR Spectroscopy and Electron Cryotomography to Study Membrane Proteins across Resolutions in Native Environments
Source: Structure. 2018 Jan 2;26(1):161–170.e3. doi: 10.1016/j.str.2017.11.011 (PMC5758107; doi:10.1016/j.str.2017.11.011)
Supplement: Document S2. Article plus Supplemental Information [file mmc2.pdf]

Resource

Structure

Combined ^{1}H-Detected Solid-State NMR Spectroscopy and Electron Cryotomography to Study Membrane Proteins across Resolutions in Native Environments

Graphical Abstract

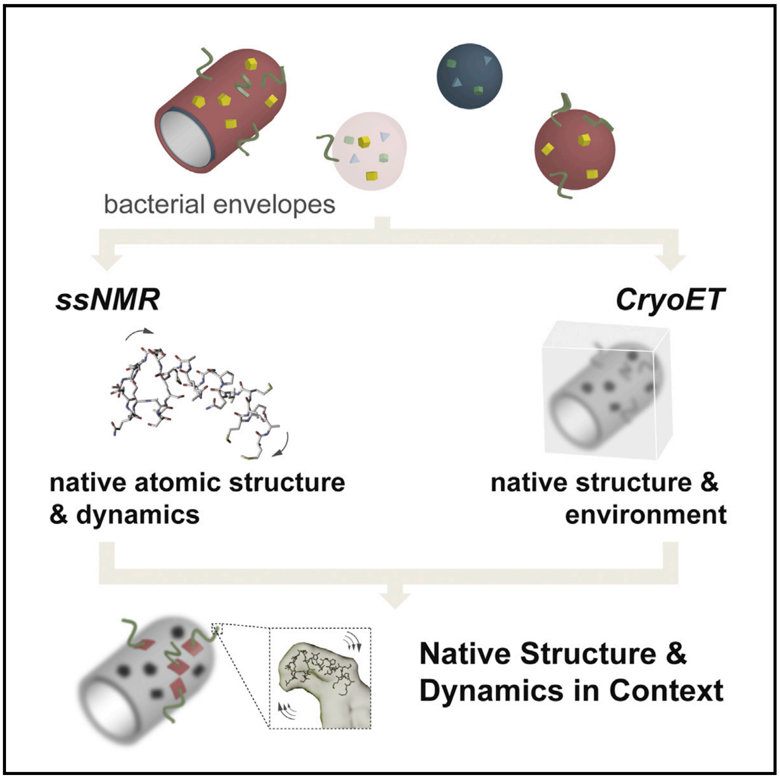

Highlights

- CryoET and ssNMR give complementary information about proteins in native membranes
- One sample can be prepared for both methods without the use of detergents
- Hybrid method shows differences between purified and native preparations of YidC
- Sample preparation reduces costs and time and suggests new strategy for assignment

Authors

Lindsay A. Baker, Tessa Sinnige, Pascale Schellenberger, ..., Arnold J.M. Driessen, Marc Baldus, Kay Grünewald

Correspondence

lindsay@strubi.ox.ac.uk (L.A.B.), m.baldus@uu.nl (M.B.), kay@strubi.ox.ac.uk (K.G.)

In Brief

Membrane proteins are often treated with detergents, which can affect structure and activity. Baker et al. apply a hybrid method to bacterial membrane proteins in native membranes without detergent, using solid-state NMR spectroscopy and electron cryotomography. They find that the structure and function of YidC differ with and without detergent.

Baker et al., 2018, Structure *26*, 161–170
January 2, 2018 © 2017 The Authors. Published by Elsevier Ltd.
https://doi.org/10.1016/j.str.2017.11.011

CellPress

CellPress

Combined ^{1}H-Detected Solid-State NMR Spectroscopy and Electron Cryotomography to Study Membrane Proteins across Resolutions in Native Environments

Lindsay A. Baker,[1,2,9,*] Tessa Sinnige,[1,5] Pascale Schellenberger,[2,6] Jeanine de Keyzer,[3,4,7] C. Alistair Siebert,[2,8] Arnold J.M. Driessen,[3,4] Marc Baldus,[1,*] and Kay Grünewald[2,*]
[1]NMR Spectroscopy, Bijvoet Center for Biomolecular Research, Utrecht University, 3584 CH Utrecht, the Netherlands
[2]Oxford Particle Imaging Centre, Division of Structural Biology, University of Oxford, The Wellcome Trust Centre for Human Genetics, Roosevelt Drive, Oxford OX3 7BN, UK
[3]Department of Molecular Microbiology, Groningen Biomolecular Sciences and Biotechnology Institute, University of Groningen, Nijenborgh 7, 9747 AG Groningen, the Netherlands
[4]The Zernike Institute for Advanced Materials, University of Groningen, Nijenborgh 11, 9747 AG Groningen, the Netherlands
[5]Present address: Centre for Misfolding Diseases, Department of Chemistry, University of Cambridge, Lensfield Road, Cambridge CB2 1EW, UK
[6]Present address: School of Life Sciences, University of Sussex, Brighton BN1 9QG, UK
[7]Present address: Hanze University of Applied Sciences, Zernikeplein 7, 9747 AS Groningen, the Netherlands
[8]Present address: Electron Bio-Imaging Centre, Diamond Light Source, Harwell Science & Innovation Campus, Didcot, Oxfordshire OX11 0DE, UK
[9]Lead Contact
*Correspondence: lindsay@strubi.ox.ac.uk (L.A.B.), m.baldus@uu.nl (M.B.), kay@strubi.ox.ac.uk (K.G.)
https://doi.org/10.1016/j.str.2017.11.011

SUMMARY

Membrane proteins remain challenging targets for structural biology, despite much effort, as their native environment is heterogeneous and complex. Most methods rely on detergents to extract membrane proteins from their native environment, but this removal can significantly alter the structure and function of these proteins. Here, we overcome these challenges with a hybrid method to study membrane proteins in their native membranes, combining high-resolution solid-state nuclear magnetic resonance spectroscopy and electron cryotomography using the same sample. Our method allows the structure and function of membrane proteins to be studied in their native environments, across different spatial and temporal resolutions, and the combination is more powerful than each technique individually. We use the method to demonstrate that the bacterial membrane protein YidC adopts a different conformation in native membranes and that substrate binding to YidC in these native membranes differs from purified and reconstituted systems.

INTRODUCTION

Cellular organization relies on compartmentalization by lipid membranes, around which cells install protein networks that establish further function. These membrane proteins are challenging to characterize, as their native environment is complex and heterogeneous. Despite significant work with membrane proteins, the rate at which new structural information is being produced has decreased since 2005 (White, 2016). Most structural techniques rely on purification of membrane proteins using detergents, followed in some cases by reconstitution into synthetic lipid bilayers. Neither system can fully mimic the complex nature of these proteins' natural environment, and the choice of mimetic can have significant impact on both structure and function (Zhou and Cross, 2013). Purification can also disrupt higher-order structure, including oligomerization (Mi et al., 2008), complex formation (Sychev et al., 1996), or metabolic organization (Schagger and Pfeiffer, 2000). Therefore, it is desirable to be able to study membrane proteins and their structure in their native membranes. Two methods that allow for structural investigations in native membranes are solid-state nuclear magnetic resonance spectroscopy (ssNMR) and electron cryotomography (cryoET).

CryoET and ssNMR provide highly complementary information. CryoET involves imaging individual events, with each molecule potentially in a different state, while ssNMR uses bulk measurements. Motion and dynamics are implicitly recorded in ssNMR experiments from nanosecond to millisecond time scales, while cryoET is limited by the speed at which samples can be vitrified, and is therefore useful for snapshots of biological processes on the seconds-to-hours timescale. Nanometer-scale spatial information is intrinsic to cryoET, while ssNMR is much more sensitive to chemical information on the Ångstrom scale. Furthermore, ssNMR exploits isotope labeling to exclude background signals, whereas cryoET records the full environment, providing macromolecular context for measurements. CryoET and ssNMR have both been used successfully to study fibrillar structures (for a review, see Cuniasse et al., 2017 and a more recent study [Gremer et al., 2017]), secretion systems accessible to *in vitro* assembly (e.g., Demers et al., 2013, 2014; Sborgi et al., 2015) and, most recently, microtubule -protein

 © 2017 The Authors. Published by Elsevier Ltd.
This is an open access article under the CC BY license (http://creativecommons.org/licenses/by/4.0/).

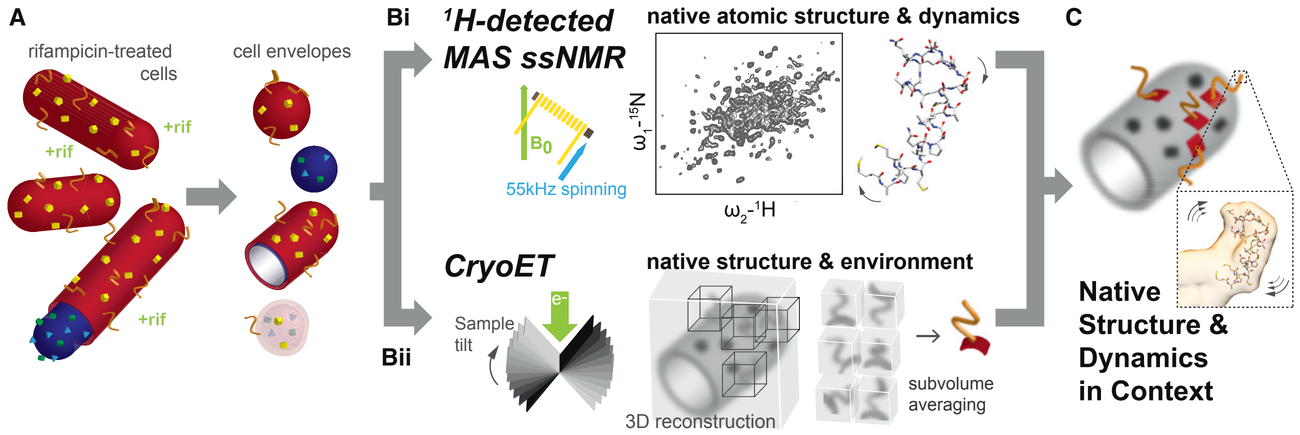

Figure 1. Overview of the Experimental Approach

(A) Isotopically labeled, rifampicin-treated cells (Baker et al., 2015) are gently disrupted to produce cell envelope particles.

(B) These cell envelope particles are subjected to (i) ^{1}H-detected magic angle spinning (MAS) solid-state NMR spectroscopy (ssNMR), which provides atomic structural and dynamical information on the protein in native membranes, and (ii) electron cryotomography (cryoET), which provides larger structural information of the protein of interest through subvolume averaging as well as the context of the native environment.

(C) Combining the information from ssNMR and cryoET allows a structural and functional model of the protein of interest and its environment to be built, from ångstrom to micron distances, in native membranes.

complexes (Atherton et al., 2017). Restraints from ssNMR experiments also recently have been proposed to aid in model refinement from electron cryomicroscopy (cryoEM) (Perilla et al., 2017).

Magic angle spinning (MAS) ssNMR (Andrew et al., 1958) is well suited to the analysis of large assemblies such as cell membranes, as it uses spinning to minimize anisotropic interactions. Conventionally, MAS with speeds of <20 kHz, in combination with ^{13}C detection, have been used to study local and overall protein structure and dynamics at atomic resolution in bilayers formed by native bacterial membranes (see, e.g., Baker and Baldus, 2014; Etzkorn et al., 2007; Herzfeld and Lansing, 2002; Hong et al., 2012; Jacso et al., 2012; Miao et al., 2012; Renault et al., 2010; Ward et al., 2015a; Yamamoto et al., 2015). These approaches have been extended to study entire bacterial cell envelopes (Kaplan et al., 2015; Renault et al., 2012a) or mammalian membrane proteins embedded in their natural plasma membrane (Kaplan et al., 2016a, 2016b). Recent methodological advancements in Dynamic Nuclear Polarization have improved spectral sensitivity for such samples (Jacso et al., 2012; Kaplan et al., 2015, 2016a, 2016b; Renault et al., 2012b; Yamamoto et al., 2015). Another area of development is in ^{1}H-detected MAS ssNMR experiments, where the higher gyromagnetic ratio of protons can enhance overall spectroscopic sensitivity provided that MAS spinning rates >40 kHz are used (Andreas et al., 2010; Asami and Reif, 2013; Medeiros-Silva et al., 2016; Sinnige et al., 2014; Ward et al., 2011). With faster spinning, line widths are generally narrower; sample preparation and choice of labels can improve spectral resolution (Andreas et al., 2010; Asami and Reif, 2013; Fricke et al., 2017; Medeiros-Silva et al., 2016; Sinnige et al., 2014; Ward et al., 2011).

CryoET has been used to study a wide range of samples, from purified protein complexes to intact viruses, bacteria, and eukaryotic cells, preserved in a frozen, hydrated state that mimics physiological conditions. Briefly, a series of projection images of the same specimen is collected with different orientations relative to the electron beam, followed by computational processing to recover three-dimensional structural information without averaging (for a recent review, see Beck and Baumeister, 2016). As the sample and stage thickness prevent tilting to 90°, there is a "missing wedge" of information in Fourier space. This missing information can be compensated for by averaging together three-dimensional subvolumes extracted from tomograms, which are differentially oriented relative to the missing wedge. CryoET (and other forms of cryoEM) also recently benefited from technological advancements. In particular, direct electron detectors have significantly increased the signal in images (McMullan et al., 2014). Some recent examples of bacterial systems studied by cryoET include work investigating the organization of the pilus in *Myxococcus xanthus* (Chang et al., 2016), the injection of pathogenic factors into host cells by *Chlamydia trachomatis* (Nans et al., 2015), and the formation of cellular structures organizing DNA replication during phage infection (Chaikeeratisak et al., 2017).

To take full advantage of the complementarity between ssNMR and cryoET, and recent technological improvements in ^{1}H detection and direct detectors, respectively, we set out to create a sample preparation method for the structural and functional study of membrane proteins in their native environment, where the same specimens could be used for both techniques. To maintain the native membrane environment, we avoided altogether the use of detergents or other extraction strategies. These samples also needed to balance the sensitivity of ^{1}H-detected ssNMR experiments with reasonable protein expression levels to avoid excess disruption to the membrane environment. As structure is tightly linked to function, accessibility to the membrane surfaces for functional or binding assays was also an important consideration. Similarly, membrane morphologies needed to be reflective of, e.g., native cell envelope ultrastructure. Furthermore, a range of orientations is desirable to compensate for the missing wedge in cryoET.

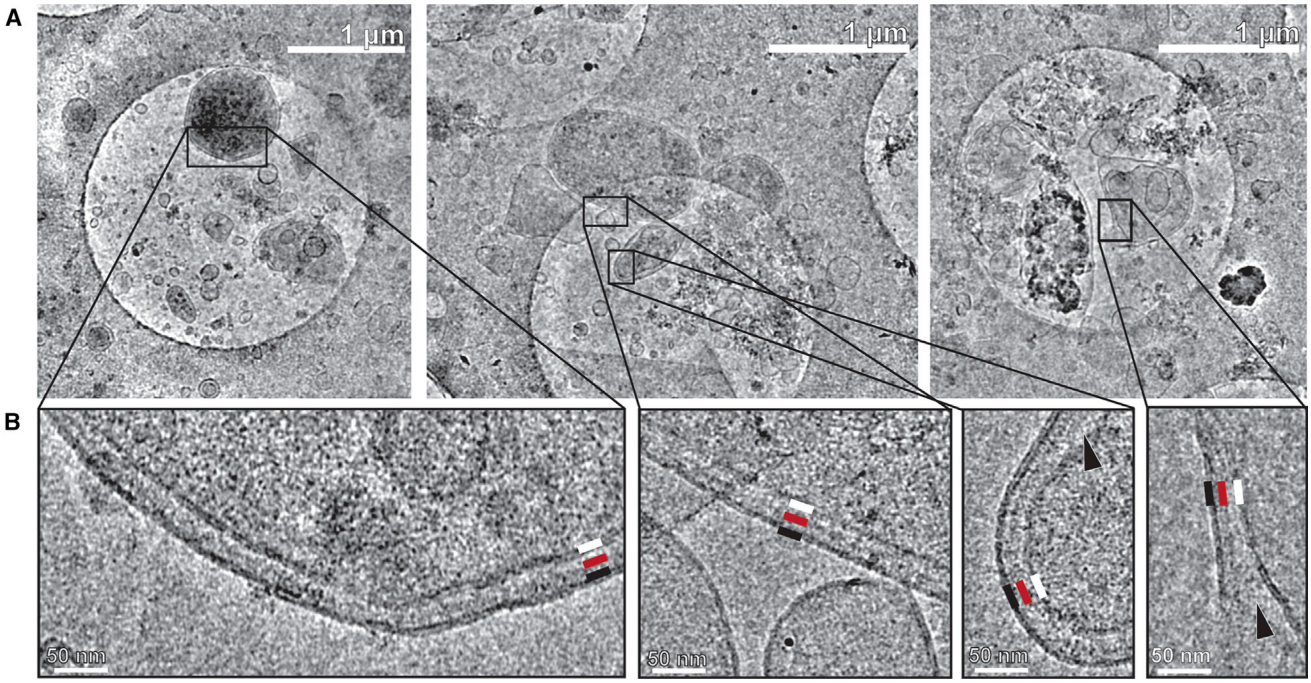

Figure 2. The Morphology of Cell Envelope Samples by cryoEM

(A) Cell envelopes were vitrified on graphene oxide-treated holey carbon grids. The membranes adopt a variety of shapes and sizes, with many maintaining a native ultrastructure.

(B) Membrane structures of different shapes and sizes maintain the native cell wall architecture when viewed at higher magnification of outer membrane (black line), cell wall (red line), and inner membrane (white line). When the inner and outer membranes separate (black arrowheads), the cell wall appears to favor the outer membranes.

See also Figures S1 and S2.

Here, we present a combined ^{1}H-detected ssNMR and cryoET investigation of the structure, function, and native environment of YidC in *Escherichia coli*. YidC is an inner membrane protein that helps fold and insert other inner membrane proteins (Scotti et al., 2000). YidC has also been shown to insert some substrates, such as subunit c of ATP synthase, independently of the Sec translocon system (van der Laan et al., 2004). *E. coli* ribosomes with substrate membrane protein nascent chains (RNCs) bind and insert substrate via purified and reconstituted YidC (Kedrov et al., 2013). The structure of purified YidC was determined in the lipidic cubic phase by X-ray crystallography (Kumazaki et al., 2014) and also, at lower resolution, in nanodiscs bound to RNCs by single-particle cryoEM (Kedrov et al., 2016). YidC can be produced with high yield after overexpression in *E. coli*, allowing for purified and reconstituted control samples (Baker et al., 2015), and binding of ribosomes to YidC has been shown to differ depending on the membrane mimetic used (Kedrov et al., 2013). We use our hybrid method to show that the conformation and likely dynamics of YidC in native membranes differs from purified and reconstituted YidC, and observe corresponding RNC binding differences.

RESULTS

Single Sample Preparation for Hybrid Methods

A key goal of this work was to develop a single sample preparation strategy for both ssNMR and cryoET. To achieve the desired sensitivity and resolution by NMR spectroscopy requires incorporation of NMR-active nuclei (^{1}H, ^{13}C, and ^{15}N) into the molecule of interest. To selectively incorporate the isotopes while maintaining an NMR-silent ^{2}H^{12}C^{14}N cell envelope background, we used the antibiotic rifampicin to inhibit the native *E. coli* RNA polymerase. The gene of interest then was transcribed by the T7 polymerase, as described previously (Baker et al., 2015). Rifampicin-treated cell envelopes have negligible background protein signal when measured by ssNMR (Baker et al., 2015). After rifampicin treatment and protein expression, cell envelopes were harvested by gentle cell lysis and ultracentrifugation. For ssNMR, the hydrated cell envelopes were centrifuged into an MAS rotor using a benchtop microfuge at low speed. For cryoET, the cell envelopes were diluted 1 in 300 in buffer before vitrification on a carbon-coated grid. An overview of the method is presented in Figure 1.

To confirm that rifampicin treatment had not changed cellular or membrane structure, we first used cryoEM to investigate the morphology of the *E. coli* cells and cell envelope samples prepared by this method. Small samples of the cultures were taken immediately before cell envelope harvesting and vitrified without any further treatment. Many cells exhibited typical size and shape (rods of ~2 μm by 1 μm), but some cells (<20%) were longer (between 3 and 12 μm) and a small number (<10%) were rounded or showed evidence of lysis (Figure S1). The appearance of the cell envelope samples also remained consistent, with or without rifampicin treatment and heterologous

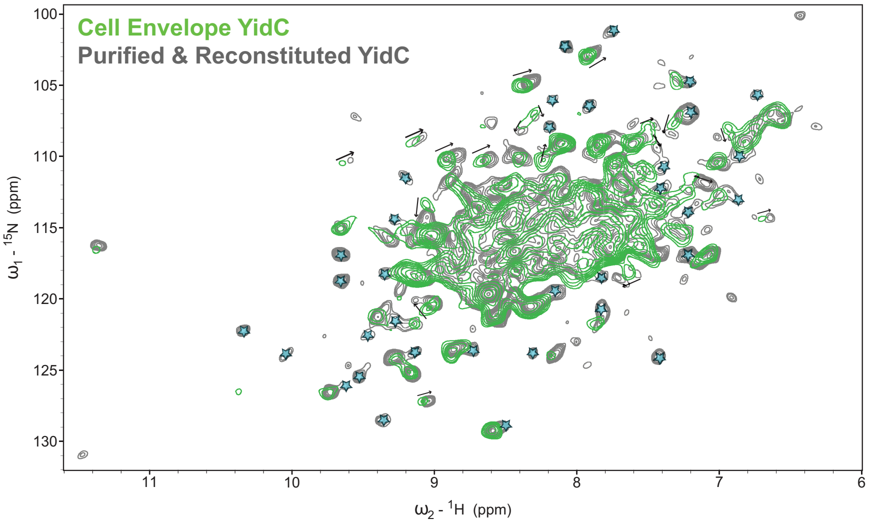

Figure 3. Overlay of ^{1}H-detected (CP-based) ^{15}N-^{1}H correlation spectra of YidC in Cell Envelopes (Green) and Purified and Reconstituted in *E. coli* Phospholipids (Gray) at 55 kHz MAS

Although overall the spectra agree well (>30 resolved peaks remain unchanged), 18 resolved peaks move (arrows) and >35 intensities change (stars) in the purified sample, relative to the cell envelope, suggesting that the structure and likely dynamics of YidC are different in native membranes. See also Figure S3.

protein expression (Figures S2A–S2C). As lysozyme was not added to the samples, larger pieces of the cell envelopes often maintained their native structure of two lipid bilayers with the cell wall between them, while smaller pieces usually consisted of a single bilayer, sometimes as a vesicle but often forming sheets or non-spherical structures (Figure 2). Attempts to form vesicles using methodologies typical for work with synthetic bilayers, such as extrusion, freeze-thawing, or sonication, produced aggregated sheet morphologies (Figure S2D), which are less suitable for electron tomography of membrane proteins.

Probing Protein Conformation and Dynamics with ^{1}H-Detected MAS ssNMR of Cell Envelope Samples

Satisfied that the YidC cell envelopes maintained a native-like ultrastructure, we then subjected them to ^{1}H-detected MAS ssNMR. For comparison, we used a conventional sample of YidC purified from the same *E. coli* strain and reconstituted into *E. coli* lipids, as described previously (Baker et al., 2015). While 1 L of ^{15}N^{13}C^{2}H culture was required to fill a 1.3-mm MAS rotor with purified and reconstituted sample, only 20 mL was required for the cell envelope sample, reducing costs by approximately 50-fold. At 55 kHz MAS and sample temperatures around 30°C, we saw no degradation in cell envelope samples treated with 2 mM EDTA for approximately 2 weeks. A two-dimensional [^{15}N,^{1}H] proton-detected Hartmann-Hahn cross-polarization (CP) experiment (Hartmann and Hahn, 1962) run on both cell envelopes and a purified and reconstituted sample is shown in Figure 3. This experiment took approximately 2–3 hr to run on an 800-MHz spectrometer for both samples; comparison of projected intensities normalized for the number of scans suggests that the concentration of YidC is about 2-fold lower in the cell envelope samples (Figure S3B). As these samples were grown in $^{2}H_2O$ and then washed in $^{1}H_2O$, only water-exchangeable protons (e.g., amide protons) will be present. Although the spectra agree in many places, there are significant differences between the native and reconstituted samples: ~18 resolvable peaks shift (mean absolute shifts of 0.05 and 0.4 ppm in ^{1}H and ^{15}N, respectively) and >35 resolvable peaks' intensities drop below the noise level (~31 resolved peaks remain relatively unchanged). Slices through the spectra near isolated peaks are shown in Figures S3C–S3F, confirming that both preparations are characterized by similar spectral resolution in our ssNMR spectra. Changes in ssNMR signal intensities may be due to changes in backbone dynamics, as CP-based (dipolar) magnetization transfer is only effective for nuclei static on microsecond timescales. Intensity variations may also result from alterations in hydrogen-deuterium exchange efficiencies, for example due to the differences in lipid composition (Ward et al., 2011), or may reflect other aspects of the complexity of cellular envelope preparations (Weingarth and Baldus, 2013). By contrast, the observed peak shifts (Figure S3G) reflect changes in the structure of YidC.

To understand these changes in structure and likely dynamics, assignment of the peaks in the spectra to specific atoms and residues in the protein is necessary. Routine assignment of larger α-helical membrane proteins remains challenging, and there are no NMR assignments available for YidC. Unfortunately, the limited agreement of chemical shift predictions precludes using the crystal structure of purified YidC as a basis for assignment (Figure S3). Significant progress has been made in obtaining ssNMR ^{13}C and ^{15}N resonance assignments for α-helical membrane proteins up to about half of the size of YidC (Cady et al., 2010; Etzkorn et al., 2007; Park et al., 2012; Sharma et al., 2010; Wang et al., 2013). Combined amino acid-specific labeling with ^{1}H ssNMR (Sinnige et al., 2014) and fractional proton labeling (Mance et al., 2015; Medeiros-Silva et al., 2016) has been used to reduce spectral complexity, but this approach is not cost effective for samples requiring large sample volumes. However, given the 50-fold reduction in sample volume for cell envelope samples, we decided to investigate amino acid-specific labeling for YidC. YidC has an extended cytoplasmic domain, which was recently shown to adopt an unusual conformation (Kumazaki et al., 2014). We therefore chose to label methionine, arginine, and lysine residues, which are prevalent in this region (Figure 4A), and avoid unintentional labeling of additional amino acids via metabolic scrambling. Out of 548 amino acids in YidC, 22 are methionine (4%), 29 are lysine (5.3%), and 15 are arginine (2.7%). One sample was prepared with methionine and arginine labeled (6.7% of total residues labeled), and another with methionine and lysine labeled (9.3% of total residues labeled). Simply by comparing two-dimensional these samples (Figures 4B–3D), we tentatively identified 10 peaks as arginine (out of 15 total),

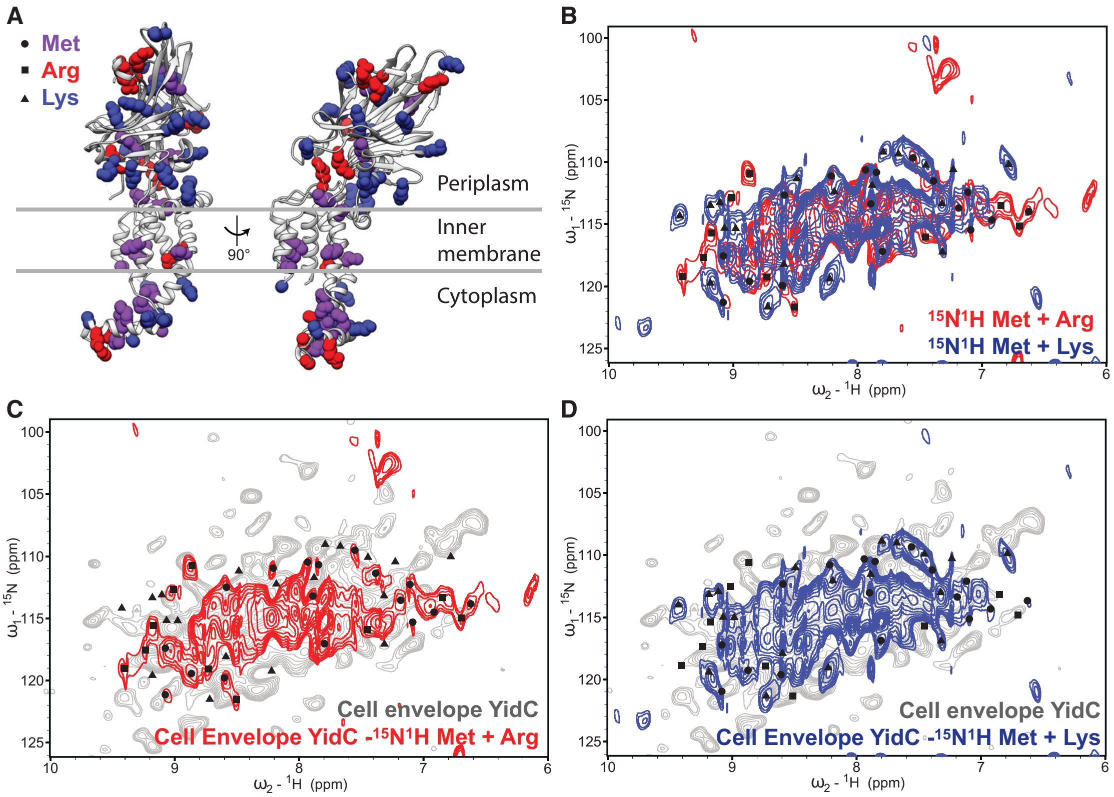

Figure 4. ^{1}H-detected ^{15}N-^{1}H Correlation Spectra of Amino Acid Specifically Labeled YidC

(A) The positions of methionine (purple), arginine (red), and lysine (blue) in the crystal structure of YidC from *E. coli* (PDB: 3VWF; Kumazaki et al., 2014), used to choose the labeling scheme for ^{15}N^{1}H CP spectra of amino acid-labeled YidC in cell envelopes.

(B) Overlay of specifically labeled spectra: red, ^{15}N^{1}H methionine and arginine; blue, ^{15}N^{1}H methionine and lysine. These amino acids are not used by *E. coli* in the metabolic production of other amino acids. By comparing the two samples, putative amino acid types (triangles, methionine; squares, arginine; circles, lysine) can be ascribed based on their presence in one (lysine or arginine, depending on sample) or both (methionine), as shown on each spectrum.

(C and D) Overlay of fully labeled YidC cell envelopes (gray) with (C) ^{15}N^{1}H methionine and arginine from (B) (red) and (D) ^{15}N^{1}H methionine and lysine from (B) (blue). All correlations seen in specifically labeled samples appear also in fully labeled YidC at lower contour levels.

19 as lysine (29 total), and 17 as methionine (22 total), demonstrating the feasibility and potential of this method. Further, the number of peaks we have identified suggests that we are observing resonances from each domain in YidC (periplasmic, transmembrane, and cytoplasmic). With improved pulse sequences for three- or higher-dimensional magnetization transfer (see, e.g., Andreas et al., 2010; Fricke et al., 2017; Ward et al., 2015b) in dynamic, heterogeneous samples such as cell envelopes, we anticipate that it will be possible to use pairwise labeling of specific amino acids to assign large α-helical membrane proteins.

Structural Characterization of Ribosomes with Nascent Chains Binding to YidC in Cell Envelope Samples by CryoET and Subvolume Averaging

YidC interacts with translating ribosomes whose nascent chain is a YidC substrate (Kedrov et al., 2013). We therefore overexpressed subunit c of ATP synthase, a YidC substrate (van der Laan et al., 2004), with an added SecM stall sequence and a *strep* tag in *E. coli* cells lacking the chaperone Trigger Factor, and purified RNCs by affinity chromatography, as described previously (Wu et al., 2012). We then incubated the RNCs with cell envelopes containing overexpressed YidC before vitrification for cryoET. Slices through an example tomogram are shown in Figure 5A. RNCs can be observed in proximity to membranes (Figures 5B and 5C). Ribosomes are known to interact with the air-water interface (Frank et al., 1991; Kedrov et al., 2016), which is typically overcome by adsorbing the ribosomes to a thin carbon film before vitrification (Frank et al., 1991), or, as recently proposed, by the addition of small amounts of detergents in the buffer (Kedrov et al., 2016). However, for our samples, adsorption to the carbon film would be as disruptive as attraction to the air-water interface, and detergent could perturb the cell envelopes, so we chose to consider only RNCs not found at the air-water interface. Of these distinct ribosomes, ~42% were in proximity to a membrane (Figure 5E).

To investigate the interaction of ribosomes with nascent chains and these membranes, we used subvolume averaging. A total of 396 subvolumes were extracted from 32 tomograms, and aligned to an initial average generated by defining the membrane normal. After several rounds of alignment, the subvolumes were classified using principal component analysis and k-means clustering on the ribosome region of the subvolumes only. Although of low resolution, the approximate position for the

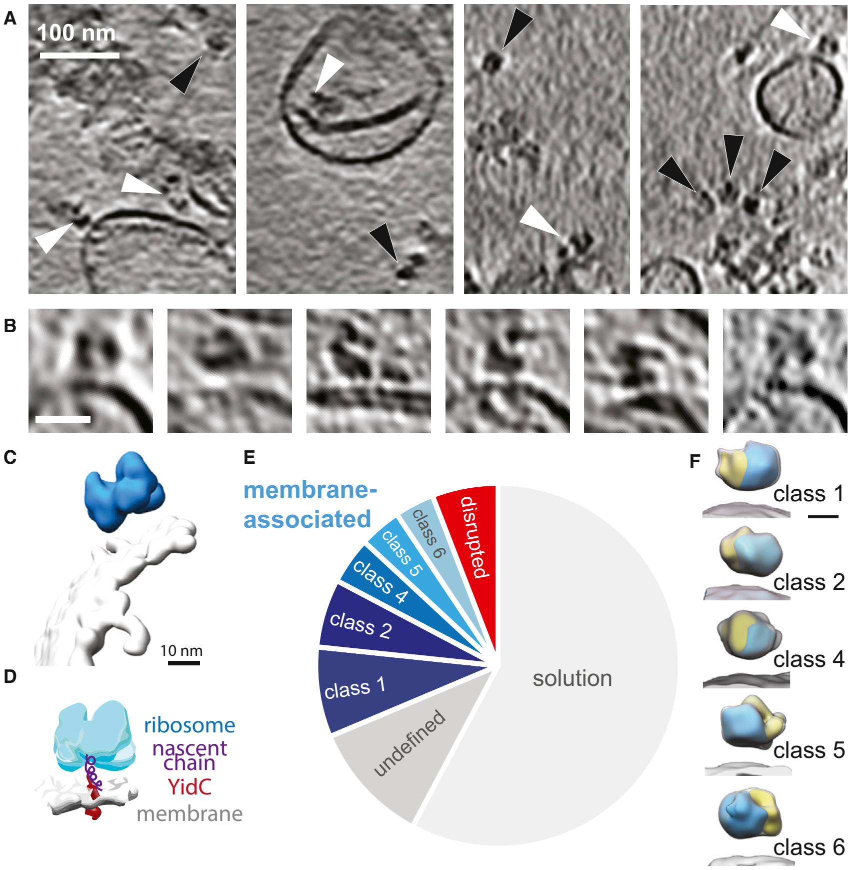

Figure 5. CryoET of YidC Cell Envelopes and Ribosomes with F_oc Nascent Chains

(A) Slices from tomograms with ratios of YidC/RNCs of 4:1. Membrane-associated RNCs are indicated with white arrowheads, while black arrowheads indicate RNCs resolved in solution.

(B) Examples of isolated RNCs associated with YidC cell envelopes. Scale bar, 25 nm.

(C) Three-dimensional representation of RNC (blue) and YidC cell envelope (gray) segmented from a tomogram using semi-automated segmentation (Baker and Rubinstein, 2011), showing the level of information available in each tomogram.

(D) Schematic showing the expected binding scheme for RNCs and YidC native membranes.

(E) Pie chart indicating the proportion of resolvable RNCs found free in solution (light gray) or associated with YidC cell envelopes. Those associated with the membrane were further subdivided by subvolume averaging and classification to eliminate any RNCs not competent for membrane binding (red, "disrupted"). Those with too few particles in their class to identify the RNC orientation were also excluded (medium gray, "undefined").

(F) Averages of the five classes of subvolumes with the RNCs in YidC-binding positions. Classes 1, 2, 4, 5, and 6 contained 75, 57, 39, 35, and 33 subvolumes, respectively. Scale bar, 10 nm.

See also Figure S4.

ribosome exit channel was identified in the averages for each class using the orientations of the large and small subunits. Eighty-two percent of the subvolumes were present in the first four classes. One class (containing ~20% of the subvolumes) was chosen as a reference for further rounds of alignment, as the ribosome in this class had an orientation that would allow its nascent chain access to the membrane to bind YidC. This alignment was followed again by classification based on the ribosome only. Five classes (containing ~60% of the subvolumes) were in YidC binding consistent orientations (Figures 5E and 5F, classes 1, 2, 4, 5, and 6). However, combining them into a single class did not improve the average. Another class (Figure 5E, "disrupted"), containing ~14% of the subvolumes, appeared to contain only large ribosomal subunits with a different orientation relative to the membrane than in the other classes (Figure S4B), and was discarded. The other classes had too few particles for sufficient signal in the average to assess their orientation (Figure 5E, "undefined"). Taking into account the number of free RNCs in solution, 27% ± 6% of RNCs were bound to the native membranes expressing YidC. Unfortunately, due to the low numbers of bound RNCs, the structures from subvolume averaging could not be refined to higher resolution.

DISCUSSION

We have established a framework for a hybrid method to probe the structure, function, and native environment of membrane proteins in native bacterial membranes using ^{1}H-detected ssNMR spectroscopy and cryoET. The combination of the two methods provides complementary information and is more powerful than each method individually. Our ssNMR data are compatible with conformational and possibly dynamical changes in YidC when comparing the native environment and reconstituted samples. In parallel, by cryoET, we observed that binding of RNCs of F_oc to YidC is different in native membranes than has been published previously for reconstituted YidC (Kedrov et al., 2016). These authors measured dissociation constants of 85 nM and 210 nM for RNC binding to YidC by fluorescence correlation spectroscopy in nanodiscs with different lipid compositions. Based on these dissociation constants, we would expect very high levels of observed RNC binding (98.9% and 97.5%, respectively) for the conditions found in the native membrane preparations here (2.5 μM [total RNC] and 10 μM [total YidC]). The role of crowding or external binding partners present in the native membranes, effects of lipid composition on RNC interactions, or our observed structural changes in YidC itself may underlie the lower binding rates for native membranes. For example, a 3-fold increase in RNC-YidC K_D was observed in the absence of DOPE (dioleoylphosphatidylethanolamine) (Kedrov et al., 2013), and the glycolipid MPIase has been proposed to modulate YidC activity (Nishikawa et al., 2017). The function of YidC suggests that less stable binding in a native environment might actually be beneficial in cells, preventing buildup of stalled ribosomes or unfolded membrane proteins and ensuring a supply of available YidC.

With native membrane preparations, by removing the use of detergent in any step of the process the biological environment of membrane proteins is maintained. We have shown that our cell envelope preparations adopt a variety of morphologies, with many maintaining the native structure of the two bilayers with a cell wall. Working with these native membranes can pose unique challenges: methods such as sonication, freeze-thawing, or extrusion, typically used for synthetic bilayers, produce different membrane morphologies when applied to cell envelopes. However, as the purification procedure is reduced substantially, the amount of time and material needed is correspondingly reduced. From transformation to the final sample, cell envelopes can be prepared in 4 days, while to purify and reconstitute often requires 2 weeks. Close to 1 L of isotopically labeled culture is needed to purify enough protein to fill a 1.3-mm MAS rotor; cell envelope samples can be grown in 20 mL, producing a significant (~50-fold) reduction in costs for isotopes with only an approximately 2-fold decrease in protein concentration. This reduction in costs opens up a new possibility for assignment of large membrane proteins by MAS ssNMR. Direct detection of protons with faster spinning speeds produces sufficient signal to observe even sparsely labeled membrane proteins in the cell envelope. Therefore, we propose that several samples, strategically labeling combinations of two or three amino acids per sample, in combination with three- or higher-dimensional experiments, provides an approach toward assignment of residues in large membrane proteins by ssNMR. This approach is significantly less intensive than mutagenesis, and can be tailored to individual proteins or domains through the choice of amino acids. Here, we have chosen to use amino acids that are not further metabolically processed in *E. coli* (Hullo et al., 2007; Jensen and Wendisch, 2013; Kanehisa et al., 2017). To avoid unintended labeling of amino acids via metabolic reprocessing where this is not the case (for example, aspartate or glutamate), $^{14}N^{12}C$ NMR-silent amino acids could be added in addition to those $^{15}N^{13}C$ amino acids to be observed in a reverse labeling strategy (Etzkorn et al., 2007; Heise et al., 2005; Umbarger and Brown, 1958; Vuister et al., 1994). Alternatively, strategically selected pairs of amino acids, where one amino acid feeds into the synthesis pathways of a second amino acid, could be labeled together.

The method presented here is generally applicable to *E. coli* membrane proteins and has the potential to be expanded to other bacterial and eukaryotic cell lines. Many bacteria are susceptible to rifampicin; a similar strategy could be used in eukaryotic cells with MazEF-based destruction of native mRNA for isotope labeling (Suzuki et al., 2005) and focused-ion beam milling to thin cells for electron tomography (Rigort et al., 2012). Future work could also focus on reducing the overexpression of the protein of interest to lower levels, more carefully mimicking the endogenous expression. Site-specific dynamic nuclear polarization could be used to increase signals in ssNMR (van der Cruijsen et al., 2015; Kaushik et al., 2016; Rogawski et al., 2017), and high-precision correlative light and electron microscopy (Schellenberger et al., 2014) could be used to locate rare events of interest in cryoET.

Conclusions

We have demonstrated a sample preparation that maintains the native environment of membrane proteins and is suitable for both ssNMR and cryoET. The use of detergents or other membrane-extraction strategies is avoided entirely, and the method can be used to probe function and binding assays with membranes reflective of the native cellular ultrastructure. By minimizing interventions, sample costs have been reduced by more than an order of magnitude, with a 3-fold decrease in preparation time. Combining cryoET and ssNMR provides complementary information: conformational and dynamical changes observed by ssNMR help interpret functional results from cryoET. This approach provides an attractive framework through which to effectively characterize the structure, dynamics, and function of membrane proteins.

STAR★METHODS

Detailed methods are provided in the online version of this paper and include the following:

- KEY RESOURCES TABLE
- CONTACT FOR REAGENTS AND RESOURCE SHARING
- EXPERIMENTAL MODEL AND SUBJECT DETAILS
- METHOD DETAILS
  - Protein Expression and Cell Envelope Isolation
  - Purified and Reconstituted Sample Preparation
  - Spectroscopy, Processing, and Referencing
  - RNC Complex Purification and Cell Envelope Binding
  - Electron Cryotomography and Image Processing
- QUANTIFICATION AND STATISTICAL ANALYSIS
- DATA AND SOFTWARE AVAILABILITY

SUPPLEMENTAL INFORMATION

Supplemental Information includes four figures and can be found with this article online at https://doi.org/10.1016/j.str.2017.11.011.

AUTHOR CONTRIBUTIONS

Conceptualization and Funding Acquisition, L.A.B., M.B., and K.G.; Methodology, L.A.B., P.S., and T.S.; Formal Analysis, Investigation, and Visualization, L.A.B.; Resources, L.A.B., J.d.K., and C.A.S.; Writing – Original Draft, L.A.B., M.B., and K.G.; Writing – Reviewing & Editing, L.A.B., M.B., K.G., T.S., P.S., J.d.K., C.A.S., and A.J.M.D.; Supervision, A.J.M.D., M.B., and K.G.

ACKNOWLEDGMENTS

We thank Mark Daniëls (Utrecht University) for supplying the YidC expression construct and Drs. T. Zeev-Ben-Mordehai and A. Baldwin for critical reading of the manuscript. We acknowledge Diamond for access and support of the cryoEM facilities at the UK National Electron Bio-Imaging Center (eBIC) (proposal EM-AP22), funded by the Wellcome Trust, MRC, and BBSRC. L.A.B. was supported by a Human Frontier Science Program Long Term Fellowship and a Canadian Institutes of Health Research Postdoctoral Fellowship. K.G. was supported by a Wellcome Trust Senior Research Fellowship (090895/Z/09/Z). This research was funded in part by NWO (grants 700.26.121 and 700.10.443 to M.B.); iNEXT (project no. 653706), a Horizon 2020 program of the European Union; a Wellcome Trust core award to The Wellcome Trust Center for Human Genetics (090532/Z/09/Z); and Project 190433215 ALW TOP to A.D.

Received: July 3, 2017
Revised: October 2, 2017
Accepted: November 15, 2017
Published: December 14, 2017

SUPPORTING CITATIONS

The following reference appears in the Supplemental Information: Taheri-Araghi et al. (2015).

REFERENCES

Andreas, L.B., Eddy, M.T., Pielak, R.M., Chou, J., and Griffin, R.G. (2010). Magic angle spinning NMR investigation of influenza A M2(18-60): support for an allosteric mechanism of inhibition. J. Am. Chem. Soc. *132*, 10958–10960.

Andrew, E.R., Bradbury, A., and Eades, R.G. (1958). Nuclear magnetic resonance spectra from a crystal rotated at high speed. Nature *182*, 1659.

Asami, S.A.M., and Reif, B. (2013). Proton-detected solid-state NMR spectroscopy at aliphatic sites: application to crystalline systems. Acc. Chem. Res. *46*, 2089–2097.

Atherton, J., Jiang, K., Stangier, M.M., Luo, Y., Houben, K., Van Hoff, J., Joseph, A.P., Scarabelli, G., Grant, B.J., Topf, M., et al. (2017). A structural model for microtubule minus-end recognition and protection by CAMSAP proteins. Nat. Struct. Mol. Biol. *24*, 931–943.

Baker, L.A., and Baldus, M. (2014). Characterization of membrane protein function by solid-state NMR spectroscopy. Curr. Opin. Struct. Biol. *27*, 48–55.

Baker, L.A., and Rubinstein, J.L. (2011). Edged watershed segmentation: a semi-interactive algorithm for segmentation of low-resolution maps from electron cryomicroscopy. J. Struct. Biol. *176*, 127–132.

Baker, L.A., Daniels, M., van der Cruijsen, E.A.W., Folkers, G.E., and Baldus, M. (2015). Efficient cellular solid-state NMR of membrane proteins by targeted protein labeling. J. Biomol. NMR *62*, 199–208.

Beck, M., and Baumeister, W. (2016). Cryo-electron tomography: can it reveal the molecular sociology of cells in atomic detail? Trends Cell Biol. *26*, 825–837.

Cady, S.D., Schmidt-Rohr, K., Wang, J., Soto, C.S., Degrado, W.F., and Hong, M. (2010). Structure of the amantadine binding site of influenza M2 proton channels in lipid bilayers. Nature *463*, 689–692.

Chaikeeratisak, V., Nguyen, K., Khanna, K., Brilot, A.F., Erb, M.L., Coker, J.K.C., Vavilina, A., Newton, G.L., Buschauer, R., Pogliano, K., et al. (2017). Assembly of a nucleus-like structure during viral replication in bacteria. Science *355*, 194–197.

Chang, Y.-W., Rettberg, L.A., Treuner-Lange, A., Iwasa, J., Søgaard-Andersen, L., and Jensen, G.J. (2016). Architecture of the type IVa pilus machine. Science *351*, aad2001.

Cuniasse, P., Tavares, P., Orlova, E.V., and Zinn-Justin, S. (2017). Structures of biomolecular complexes by combination of NMR and cryoEM methods. Curr. Opin. Struct. Biol. *43*, 104–113.

Demers, J.-P., Sgourakis, N.G., Gupta, R., Loquet, A., Giller, K., Riedel, D., Laube, B., Kolbe, M., Baker, D., Becker, S., et al. (2013). The common structural architecture of *Shigella flexneri* and *Salmonella typhimurium* type three secretion needles. PLoS Pathog. *9*, e1003245.

Demers, J.-P., Habenstein, B., Loquet, A., Kumar Vasa, S., Giller, K., Becker, S., Baker, D., Lange, A., and Sgourakis, N.G. (2014). High-resolution structure of the *Shigella* type-III secretion needle by solid-state NMR and cryo-electron microscopy. Nat. Commun. *5*, 4976.

Etzkorn, M., Martell, S., Andronesi, O.C., Seidel, K., Engelhard, M., and Baldus, M. (2007). Secondary structure, dynamics, and topology of a seven-helix receptor in native membranes, studied by solid-state NMR spectroscopy. Angew. Chem. Int. Ed. *46*, 459–462.

Folkers, G.E., van Buuren, B.N.M., and Kaptein, R. (2004). Expression screening, protein purification and NMR analysis of human protein domains for structural genomics. J. Struct. Funct. Genomics *5*, 119–131.

Frank, J., Penczek, P., Grassucci, R., and Srivastava, S. (1991). Three-dimensional reconstruction of the 70S *Escherichia coli* ribosome in ice: the distribution of ribosomal RNA. J. Cell Biol. *115*, 597–605.

Fricke, P., Chevelkov, V., Zinke, M., Giller, K., Becker, S., and Lange, A. (2017). Backbone assignment of perdeuterated proteins by solid-state NMR using proton detection and ultrafast magic-angle spinning. Nat. Protoc. *12*, 764–782.

Goddard, T.D., and Kneller, D.G. (2008). SPARKY 3 (University of California, San Francisco).

Gradmann, S., Ader, C., Heinrich, I., Nand, D., Dittmann, M., Cukkemane, A., van Dijk, M., Bonvin, A.M.J.J., Engelhard, M., and Baldus, M. (2012). Rapid prediction of multi-dimensional NMR data sets. J. Biomol. NMR *54*, 377–387.

Grant, T., and Grigorieff, N. (2015). Measuring the optimal exposure for single particle cryo-EM using a 2.6 A reconstruction of rotavirus VP6. Elife *4*, e06980.

Gremer, L., Schölzel, D., Schenk, C., Reinartz, E., Labahn, J., Ravelli, R.B.G., Tusche, M., Lopez-Iglesias, C., Hoyer, W., Heise, H., et al. (2017). Fibril structure of amyloid-ß(1-42) by cryoelectron microscopy. Science *358*, 116–119.

Han, B., Liu, Y., Ginzinger, S.W., and Wishart, D.S. (2011). SHIFTX2: significantly improved protein chemical shift prediction. J. Biomol. NMR *50*, 43.

Harris, R.K., Becker, E.D., Cabral de Menezes, S.M., Granger, P., Hoffman, R.E., and Zilm, K.W. (2008). Further conventions for NMR shielding and chemical shifts (IUPAC Recommendations 2008). Pure Appl. Chem. *80*, 59–84.

Hartmann, S.R., and Hahn, E.L. (1962). Nuclear double resonance in the rotating frame. Phys. Rev. *128*, 2042–2053.

Heise, H., Hoyer, W., Becker, S., Andronesi, O.C., Riedel, D., and Baldus, M. (2005). Molecular-level secondary structure, polymorphism, and dynamics of full-length alpha-synuclein fibrils studied by solid-state NMR. Proc. Natl. Acad. Sci. USA *102*, 15871–15876.

Herzfeld, J., and Lansing, J.C. (2002). Magnetic resonance studies of the bacteriorhodopsin pump cycle. Annu. Rev. Biophys. Biomol. Struct. *31*, 73–95.

Heumann, J.M., Hoenger, A., and Mastronarde, D.N. (2011). Clustering and variance maps for cryo-electron tomography using wedge-masked differences. J. Struct. Biol. *175*, 288–299.

Hong, M., Zhang, Y., and Hu, F. (2012). Membrane protein structure and dynamics from NMR Spectroscopy. Annu. Rev. Phys. Chem. *63*, 1–24.

Hullo, M.F., Auger, S., Soutourina, O., Barzu, O., Yvon, M., Danchin, A., and Martin-Verstraete, I. (2007). Conversion of methionine to cysteine in Bacillus subtilis and its regulation. J. Bacteriol. *189*, 187–197.

Jacso, T., Franks, W.T., Rose, H., Fink, U., Broecker, J., Keller, S., Oschkinat, H., and Reif, B. (2012). Characterization of membrane proteins in isolated native cellular membranes by dynamic nuclear polarization solid-state NMR spectroscopy without purification and reconstitution. Angew. Chem. Int. Ed. *51*, 432–435.

Jensen, J.V.K., and Wendisch, V.F. (2013). Ornithine cyclodeaminase-based proline production by *Corynebacterium glutamicum*. Microb. Cell Fact. *12*, 63.

Kanehisa, M., Furumichi, M., Tanabe, M., Sato, Y., and Morishima, K. (2017). KEGG: new perspectives on genomes, pathways, diseases and drugs. Nucleic Acids Res. *45*, D353–D361.

Kaplan, M., Cukkemane, A., van Zundert, G.C.P., Narasimhan, S., Daniels, M., Mance, D., Waksman, G., Bonvin, A.M.J.J., Fronzes, R., Folkers, G.E., et al. (2015). Probing a cell-embedded megadalton protein complex by DNP-supported solid-state NMR. Nat. Methods *12*, 649–652.

Kaplan, M., Narasimhan, S., de Heus, C., Mance, D., van Doorn, S., Houben, K., Popov-Čeleketić, D., Damman, R., Katrukha, E.A., Jain, P., et al. (2016a). EGFR dynamics change during activation in native membranes as revealed by NMR. Cell *167*, 1241–1251.e11.

Kaplan, M., Pinto, C., Houben, K., and Baldus, M. (2016b). Nuclear magnetic resonance (NMR) applied to membrane-protein complexes. Q. Rev. Biophys. *49*, e15.

Kaushik, M., Bahrenberg, T., Can, T.V., Caporini, M.A., Silvers, R., Heiliger, J., Smith, A.A., Schwalbe, H., Griffin, R.G., and Corzilius, B. (2016). Gd(iii) and Mn(ii) complexes for dynamic nuclear polarization: small molecular chelate polarizing agents and applications with site-directed spin labeling of proteins. Phys. Chem. Chem. Phys. *18*, 27205–27218.

Kedrov, A., Sustarsic, M., de Keyzer, J., Caumanns, J.J., Wu, Z.C., and Driessen, A.J.M. (2013). Elucidating the native architecture of the YidC: ribosome complex. J. Mol. Biol. *425*, 4112–4124.

Kedrov, A., Wickles, S., Crevenna, A.H., van der Sluis, E.O., Buschauer, R., Berninghausen, O., Lamb, D.C., and Beckmann, R. (2016). Structural

dynamics of the YidC: ribosome complex during membrane protein biogenesis. Cell Rep. *17*, 2943–2954.

Kremer, J.R., Mastronarde, D.N., and McIntosh, J.R. (1996). Computer visualization of three-dimensional image data using IMOD. J. Struct. Biol. *116*, 71–76.

Kumazaki, K., Kishimoto, T., Furukawa, A., Mori, H., Tanaka, Y., Dohmae, N., Ishitani, R., Tsukazaki, T., and Nureki, O. (2014). Crystal structure of *Escherichia coli* YidC, a membrane protein chaperone and insertase. Sci. Rep. *4*, 7299.

van der Laan, M., Bechtluft, P., Kol, S., Nouwen, N., and Driessen, A.J.M. (2004). F1F0 ATP synthase subunit c is a substrate of the novel YidC pathway for membrane protein biogenesis. J. Cell Biol. *165*, 213–222.

Mance, D., Sinnige, T., Kaplan, M., Narasimhan, S., Daniels, M., Houben, K., Baldus, M., and Weingarth, M. (2015). An efficient labelling approach to harness backbone and side-chain protons in (1)H-detected solid-state NMR spectroscopy. Angew. Chem. Int. Ed. *54*, 15799–15803.

Mastronarde, D.N. (2005). Automated electron microscope tomography using robust prediction of specimen movements. J. Struct. Biol. *152*, 36–51.

McMullan, G., Faruqi, A.R., Clare, D., and Henderson, R. (2014). Comparison of optimal performance at 300keV of three direct electron detectors for use in low dose electron microscopy. Ultramicroscopy *147*, 156–163.

Medeiros-Silva, J., Mance, D., Daniels, M., Jekhmane, S., Houben, K., Baldus, M., and Weingarth, M. (2016). ^{1}H-detected solid-state NMR studies of water-inaccessible proteins in vitro and in situ. Angew. Chem. Int. Ed. *55*, 13606–13610.

Mi, L.-Z., Grey, M.J., Nishida, N., Walz, T., Lu, C., and Springer, T.A. (2008). Functional and structural stability of the epidermal growth factor receptor in detergent micelles and phospholipid nanodiscs. Biochemistry *47*, 10314–10323.

Miao, Y., Qin, H., Fu, R., Sharma, M., Can, T.V., Hung, I., Luca, S., Gor'kov, P.L., Brey, W.W., and Cross, T.A. (2012). M2 proton channel structural validation from full-length protein samples in synthetic bilayers and *E. coli* membranes. Angew. Chem. Int. Ed. *51*, 8383–8386.

Nans, A., Kudryashev, M., Saibil, H.R., and Hayward, R.D. (2015). Structure of a bacterial type III secretion system in contact with a host membrane in situ. Nat. Commun. *6*, 10114.

Nishikawa, H., Sasaki, M., and Nishiyama, K. (2017). Membrane insertion of F_0 c subunit of F_0F_1 ATPase depends on glycolipozyme MPIase and is stimulated by YidC. Biochem. Biophys. Res. Commun. *487*, 477–482.

Park, S.H., Das, B.B., Casagrande, F., Tian, Y., Nothnagel, H.J., Chu, M., Kiefer, H., Maier, K., De Angelis, A.A., Marassi, F.M., et al. (2012). Structure of the chemokine receptor CXCR1 in phospholipid bilayers. Nature *491*, 779–783.

Perilla, J.R., Zhao, G., Lu, M., Ning, J., Hou, G., Byeon, I.-J.L., Gronenborn, A.M., Polenova, T., and Zhang, P. (2017). CryoEM structure refinement by integrating NMR chemical shifts with molecular dynamics simulations. J. Phys. Chem. B *121*, 3853–3863.

Pettersen, E.F., Goddard, T.D., Huang, C.C., Couch, G.S., Greenblatt, D.M., Meng, E.C., and Ferrin, T.E. (2004). UCSF chimera—a visualization system for exploratory research and analysis. J. Comput. Chem. *25*, 1605–1612.

Renault, M., Cukkemane, A., and Baldus, M. (2010). Solid-state NMR spectroscopy on complex biomolecules. Angew. Chem. Int. Ed. *49*, 8346–8357.

Renault, M., Tommassen-van Boxtel, R., Bos, M.P., Post, J.A., Tommassen, J., and Baldus, M. (2012a). Cellular solid-state nuclear magnetic resonance spectroscopy. Proc. Natl. Acad. Sci. USA *109*, 4863–4868.

Renault, M., Pawsey, S., Bos, M.P., Koers, E.J., Nand, D., Tommassen-van Boxtel, R., Rosay, M., Tommassen, J., Maas, W.E., and Baldus, M. (2012b). Solid-state NMR spectroscopy on cellular preparations enhanced by dynamic nuclear polarization. Angew. Chem. Int. Ed. *51*, 2998–3001.

Rigort, A., Bauerlein, F.J.B., Villa, E., Eibauer, M., Laugks, T., Baumeister, W., and Plitzko, J.M. (2012). Focused ion beam micromachining of eukaryotic cells for cryoelectron tomography. Proc. Natl. Acad. Sci. USA *109*, 4449–4454.

Rogawski, R., Sergeyev, I.V., Li, Y., Ottaviani, M.F., Cornish, V., and McDermott, A.E. (2017). Dynamic nuclear polarization signal enhancement with high-affinity biradical tags. J. Phys. Chem. B *121*, 1169–1175.

Rutkowska, A., Mayer, M.P., Hoffmann, A., Merz, F., Zachmann-Brand, B., Schaffitzel, C., Ban, N., Deuerling, E., and Bukau, B. (2008). Dynamics of trigger factor interaction with translating ribosomes. J. Biol. Chem. *283*, 4124–4132.

Sborgi, L., Ravotti, F., Dandey, V.P., Dick, M.S., Mazur, A., Reckel, S., Chami, M., Scherer, S., Huber, M., Bockmann, A., et al. (2015). Structure and assembly of the mouse ASC inflammasome by combined NMR spectroscopy and cryo-electron microscopy. Proc. Natl. Acad. Sci. USA *112*, 13237–13242.

Schagger, H., and Pfeiffer, K. (2000). Supercomplexes in the respiratory chains of yeast and mammalian mitochondria. EMBO J. *19*, 1777–1783.

Schellenberger, P., Kaufmann, R., Siebert, C.A., Hagen, C., Wodrich, H., and Grunewald, K. (2014). High-precision correlative fluorescence and electron cryo microscopy using two independent alignment markers. Ultramicroscopy *143*, 41–51.

Schindelin, J., Arganda-Carreras, I., Frise, E., Kaynig, V., Longair, M., Pietzsch, T., Preibisch, S., Rueden, C., Saalfeld, S., Schmid, B., et al. (2012). Fiji: an open-source platform for biological-image analysis. Nat. Methods *9*, 676–682.

Scotti, P.A., Urbanus, M.L., Brunner, J., de Gier, J.W., von Heijne, G., van der Does, C., Driessen, A.J., Oudega, B., and Luirink, J. (2000). YidC, the *Escherichia coli* homologue of mitochondrial Oxa1p, is a component of the Sec translocase. EMBO J. *19*, 542–549.

Sharma, M., Yi, M., Dong, H., Qin, H., Peterson, E., Busath, D.D., Zhou, H.-X., and Cross, T.A. (2010). Insight into the mechanism of the influenza a proton channel from a structure in a lipid bilayer. Science *330*, 509–512.

Sinnige, T., Daniels, M., Baldus, M., and Weingarth, M. (2014). Proton clouds to measure long-range contacts between nonexchangeable side chain protons in solid-state NMR. J. Am. Chem. Soc. *136*, 4452–4455.

Suzuki, M., Zhang, J., Liu, M., Woychik, N.A., and Inouye, M. (2005). Single protein production in living cells facilitated by an mRNA interferase. Mol. Cell *18*, 253–261.

Sychev, S.V., Sukhanov, S.V., Barsukov, L.I., and Ivanov, V.T. (1996). Structural polymorphism of gramicidin A channels: ion conductivity and spectral studies. J. Pept. Sci. *2*, 141–156.

Taheri-Araghi, S., Bradde, S., Sauls, J.T., Hill, N.S., Levin, P.A., Paulsson, J., Vergassola, M., and Jun, S. (2015). Cell-size control and homeostasis in bacteria. Curr. Biol. *25*, 385–391.

Umbarger, H.E., and Brown, B. (1958). Isoleucine and valine metabolism in *Escherichia coli* VII. A negative feedback mechanism of controlling isoleucine biosynthesis. J. Biochem. *233*, 415–420.

van der Cruijsen, E.A.W., Koers, E.J., Sauvée, C., Hulse, R.E., Weingarth, M., Ouari, O., Perozo, E., Tordo, P., and Baldus, M. (2015). Biomolecular DNP-supported NMR spectroscopy using site-directed spin labeling. Chemistry *21*, 12971–12977.

Vuister, G.W., Kim, S.J., Wu, C., and Bax, A. (1994). 2D and 3D NMR-study of phenylalanine residues in proteins by reverse isotopic labeling. J. Am. Chem. Soc. *116*, 9206–9210.

Wang, S., Munro, R.A., Shi, L., Kawamura, I., Okitsu, T., Wada, A., Kim, S.-Y., Jung, K.-H., Brown, L.S., and Ladizhansky, V. (2013). Solid-state NMR spectroscopy structure determination of a lipid-embedded heptahelical membrane protein. Nat. Methods *10*, 1007–1012.

Ward, M.E., Shi, L., Lake, E., Krishnamurthy, S., Hutchins, H., Brown, L.S., and Ladizhansky, V. (2011). Proton-detected solid-state NMR reveals intramembrane polar networks in a seven-helical transmembrane protein proteorhodopsin. J. Am. Chem. Soc. *133*, 17434–17443.

Ward, M.E., Wang, S., Munro, R., Ritz, E., Hung, I., Gor'kov, P.L., Jiang, Y., Liang, H., Brown, L.S., and Ladizhansky, V. (2015a). In situ structural studies of *Anabaena* sensory rhodopsin in the E. coli membrane. Biophys. J. *108*, 1683–1696.

Ward, M.E., Brown, L.S., and Ladizhansky, V. (2015b). Advanced solid-state NMR techniques for characterization of membrane protein structure and dynamics: application to *Anabaena* sensory rhodopsin. J. Magn. Reson. *253*, 119–128.

Wei, Y., de Dios, A.C., and McDermott, A.E. (1999). Solid-State ^{15}N NMR chemical shift anisotropy of histidines: experimental and theoretical studies of hydrogen bonding. J. Am. Chem. Soc. *121*, 10389–10394.

Weingarth, M., and Baldus, M. (2013). Solid-state NMR-based approaches for supramolecular structure elucidation. Acc. Chem. Res. *46*, 2037–2046.

White, S. (2016). Membrane Proteins of known 3D Structure. http://blanco.biomol.uci.edu/mpstruc/.

Wu, Z.C., de Keyzer, J., Kedrov, A., and Driessen, A.J.M. (2012). Competitive binding of the SecA ATPase and ribosomes to the SecYEG translocon. J. Biol. Chem. *287*, 7885–7895.

Wu, Z.C., de Keyzer, J., Berrelkamp-Lahpor, G.A., and Driessen, A.J.M. (2013). Interaction of Streptococcus mutans YidC1 and YidC2 with translating and nontranslating ribosomes. J. Bacteriol. *195*, 4545–4551.

Yamamoto, K., Caporini, M.A., Im, S.-C., Waskell, L., and Ramamoorthy, A. (2015). Cellular solid-state NMR investigation of a membrane protein using dynamic nuclear polarization. Biochim. Biophys. Acta *1848*, 342–349.

Zhou, H.-X., and Cross, T.A. (2013). Influences of membrane mimetic environments on membrane protein structures. Annu. Rev. Biophys. *42*, 361–392.

STAR★METHODS

KEY RESOURCES TABLE

REAGENT or RESOURCE	SOURCE	IDENTIFIER
Bacterial and Virus Strains		
Escherichia coli LEMO	New England Biolabs	C2528J
Escherichia coli BL21 (DE3) ΔTrigger Factor	(Rutkowska et al., 2008)	N/A
Chemicals, Peptides, and Recombinant Proteins		
$^{13}C^{2}H$ glucose	Sigma-Aldrich	552151
$^{15}N^{1}H_4Cl_3$	Sigma-Aldrich	299251
$^{15}N^{13}C^{1}H$ lysine	Sigma-Aldrich	608041
$^{15}N^{13}C^{1}H$ arginine	Sigma-Aldrich	608033
$^{15}N^{13}C^{1}H$ methionine	Sigma-Aldrich	608106
rifampicin	Sigma-Aldrich	R3501
Deuterium oxide	Sigma-Aldrich	151882
Critical Commercial Assays		
Pierce BCA Protein Assay Kit	Thermo Fisher	23225
Deposited Data		
Native membrane YidC-RNC density map, average all	This paper	EMDB - 3909
Native membrane YidC-RNC density map, class 1	This paper	EMDB – 3919
Native membrane YidC-RNC density map, class 2	This paper	EMDB – 3920
Native membrane YidC-RNC density map, class 3	This paper	EMDB - 3921
Native membrane YidC-RNC density map, class 4	This paper	EMDB - 3922
Native membrane YidC-RNC density map, class 5	This paper	EMDB - 3923
Native membrane YidC-RNC density map, class 6	This paper	EMDB - 3924
Recombinant DNA		
pJK763	(Wu et al., 2013)	N/A
pHisLIC_YidC	(Baker et al., 2015)	N/A
Software and Algorithms		
IMOD	(Kremer et al., 1996)	http://bio3d.colorado.edu/imod/
Peet	(Heumann et al., 2011)	http://bio3d.colorado.edu/PEET/
Chimera	(Pettersen et al., 2004)	https://www.cgl.ucsf.edu/chimera/
Topspin	Bruker Biospin	https://www.bruker.com/service/support-upgrades/software-downloads/nmr.html
Sparky	(Goddard and Kneller, 2008)	https://www.cgl.ucsf.edu/home/sparky/
SerialEM	(Mastronarde, 2005)	http://bio3d.colorado.edu/SerialEM/
Tomography	Thermo Fisher (FEI)	https://www.fei.com/software/tomography-4/
Other		
Quantifoil holey carbon copper grids	Quantifoil Micro Tools GmbH	Cu 200 R2/1
Graphene oxide	Sigma-Aldrich	763705

CONTACT FOR REAGENTS AND RESOURCE SHARING

Further information and requests for resources and reagents should be directed to and will be fulfilled by the Lead Contact, Lindsay Baker (lindsay@strubi.ox.ac.uk).

EXPERIMENTAL MODEL AND SUBJECT DETAILS

Escherichia coli LEMO (New England Biolabs) was cultured in LB (10 g/L tryptone, 10 g/L NaCl, 5 g/L yeast extract, pH 7.0) or M9 minimal medium (6 g/L Na_2HPO_4, 3 g/L KH_2PO_4, 0.5 g/L NaCl, 1 g/L NH_4Cl, 5 g/L glucose, 5.5 mg/L thiamine, 0.01 mM

$FeSO_4$; 2 mM $MgSO_4$; 0.01 mM $CaCl_2$; micronutrients: 0.003 μM ammonium molybdate, 0.4 μM boric acid, 0.03 μM cobalt chloride, 0.01 μM copper sulphate, 0.08 μM manganese chloride, 0.01 μM zinc chloride; and vitamins: 1 mg/L D-biotin, 0.5 mg/L choline chloride, 0.5 mg/L folic acid, 1 mg/L myoinositol, 0.5 mg/L nicatinamide, 0.5 mg/L panthotenic acid, 0.5 mg/L pyridoxal HCl, 0.05 mg/L riboflavin) with 35 μg/ml chloramphenicol at 37 °C unless otherwise specified. Single colonies were selected on LB agar, and liquid cultures were grown with 250 rpm shaking.

METHOD DETAILS

Protein Expression and Cell Envelope Isolation

A previously described plasmid (Baker et al., 2015) carrying the gene *yidC* from *Escherichia coli* (UniProt ID: P25714) with a N-terminal 6xHis tag under a T7 promoter was used to transform *E. coli* LEMO cells (New England Biolabs) by heat shock before plating on LB with ampicillin (AMP) at 50 μg/ml and chloramphenicol (CAM) at 35 μg/ml. Precultures were grown in LB with AMP and CAM and then transferred to the M9 minimal media described above (Folkers et al., 2004) with 1.0 g/L $^{14}NH_4Cl$, 5.0 g/L $^{12}C_6$-glucose, AMP and CAM overnight. Between 50 and 500 mL volumes of M9, supplemented as above but with $^{12}C^2H$-glucose and prepared in D_2O, were inoculated with the overnight cultures to OD 0.1, and grown at 37 °C with shaking until reaching an OD ~ 1.5 - 2.0. Cells were harvested by centrifugation at 4,000 x g for 15 minutes before resuspension in equal volumes isotopically labeled supplemented M9 (1.0 g/L $^{15}NH_4Cl$, 5.0 g/L $^{13}C^2H$-glucose for uniformly labeled samples in D_2O; 1.0 g/L $^{14}NH_4Cl$, 5.0 g/L $^{12}C^2H$-glucose and 200 mg/L each ^1H,^{15}N and ^{13}C labeled amino acid in D_2O for specifically labeled samples). IPTG (isopropyl β-D-1-thiogalactopyranoside) at 0.5-1 mM final concentration was added to induce expression of the T7 polymerase and the cultures were incubated at 28 °C for 30 min with shaking. Rifampicin was added to a final concentration of 100 μg/mL and the cultures were incubated with shaking in the dark at 25 °C overnight. Cells were harvested by centrifugation at 4,000 x g for 10 min at 4 °C, and resuspended in 10 mL cold lysis buffer (20 mM Tris pH 7.4, 150 mM NaCl in H_2O). Cells were lysed in a pressure cell homogenizer (Stansted) or constant flow cell disruptor (Constant Systems) at 8,000 psi without the addition of lysozyme. Cell debris was removed by centrifugation at 4,000 x g for 10 min. Membranes were harvested by centrifugation at 100,000 x g for 1 hr.

Purified and Reconstituted Sample Preparation

Reference YidC proteoliposome samples were prepared in a similar manner to the cell envelope samples, as described previously (Baker et al., 2015). Briefly, after preculture, the main culture was inoculated in M9 in D_2O with $^{13}C^2H$-glucose and $^{15}NH_4Cl$ at OD 0.1, and grown to OD 0.6 prior to induction with IPTG and 10 μM final concentration rhamnose. Cells were grown at 37 °C until the OD was greater than 2.0, then pelleted and lysed, and the membranes isolated as described for the cell envelope samples. Membranes were solubilized at 4 °C by stirring with 2 % (w/v) dodecyl maltoside (DDM) (Anatrace) in 10 mM phosphate buffer (pH 6.8) containing 250 mM NaCl, 2 mM 2-mercaptoethanol, 10 % (v/v) glycerol, 0.03 % (w/v) DDM and 20 mM imidazole (Buffer A) for 2 hours. Insoluble material was pelleted at 100,000 xg for 1 hr, and the supernatant was bound to 5 mL Ni-NTA resin (Qiagen) overnight. The resin was washed with 4 column volumes (CV) of buffer A, and then with 2 CV of buffer B (10 mM phosphate buffer pH 6.8, 100 mM NaCl, 2 mM 2-mercaptoethanol, 10 % (v/v) glycerol, 0.03 % (w/v) DDM and 20 mM imidazole). YidC was eluted with 5 CV of buffer B with 380 mM additional imidazole. The eluent was dialyzed four times for ~2 hrs against 75 mL of Buffer B without imidazole. The protein concentration was estimated using the BCA assay (Pierce) and then mixed with *E. coli* polar lipid extract (Avanti Polar Lipids), dissolved in water, at a ratio of 2 mg YidC: 1 mg lipid. DDM was removed by overnight incubation with BioBeads (BioRad) and YidC proteoliposomes were harvested by centrifugation at 100,000 xg for 1 hour. The proteoliposomes were washed in 10 mM phosphate buffer, pH 6.8, and pelleted at 125,000 xg for ~ 2hrs before being packed into a 1.3 mm MAS rotor.

Spectroscopy, Processing, and Referencing

Membranes were washed twice with 10 mM phosphate buffer pH 6.8 (in H_20) to remove any Tris from the lysis buffer and collected by centrifugation at 125,000 xg for 1 – 2 hrs before packing into a 1.3 mm rotor for magic angle spinning (MAS). YidC samples were measured on a 800 MHz wide-bore or 700 MHz narrow-bore spectrometer (Bruker Biospin, Germany) with a 1.3 mm ^1H, ^{13}C, ^{15}N MAS probe at 55 kHz MAS frequency, and with a set temperature of 253 K (corresponding to an effective temperature of ~ 30 °C). Spectra were referenced against adamantane (Harris et al., 2008) and histidine (Wei et al., 1999) powders. Data were processed with TopSpin 3.0 (Bruker Biospin) and analyzed using Sparky (Goddard and Kneller, 2008). Chemical shift predictions were made with Shiftx2 (Han et al., 2011) and FANDAS (Gradmann et al., 2012) using atomic model PDB ID 3wvf (Kumazaki et al., 2014).

RNC Complex Purification and Cell Envelope Binding

Ribosomes with F_oc nascent chains (RNCs) were prepared as described previously (Wu et al., 2012). Briefly, F_oc with a SecM stall sequence and *strep* tag was over-expressed in an *E. coli* strain lacking the chaperone Trigger Factor. Cells were cooled on ice and harvested by centrifugation, before repeated cycles of freeze-thaw to lyse. Cell debris was removed by centrifugation at 4000xg for 10 min and RNCs were purified by affinity chromatography using streptactin beads (IBA Bioscience), eluted with biotin, and concentrated to 2 uM with centrifugal concentrators (Millipore) before snap freezing in liquid nitrogen and storage at -80°C in aliquots until further use. For RNC binding experiments, membranes were diluted 1:30 in 50 mM Tris-HCl pH 7.4, 150 mM KCl, 10 mM $MgCl_2$ before 10 ul was mixed with 10 ul of RNC and incubated on ice for 1 hr. The mixture was diluted 1:10 in the same buffer before

vitrifying as described for cell envelopes without RNCs below. Final concentrations of RNCs and YidC were estimated using the tomogram volumes and assuming a uniform distribution of YidC in the *E. coli* inner membrane.

Electron Cryotomography and Image Processing

Cell envelopes were diluted 1:300 from the ssNMR samples in lysis buffer before application to Quantifoil 2/1 holey carbon grids with or without the addition of graphene oxide. Graphene oxide grids were prepared immediately before use by applying 2 ul of a 2 mg/ml solution of graphene oxide sheets (Sigma) to a freshly glow-discharged grid for 30 s, blotting away excess, and washing three times in water. Samples were incubated on grids for 10 s, before for blotting for 8 s by hand and plunging into a bath of propane/ethane with a manual plunger. Grids were stored under liquid nitrogen until imaging. Electron microscopy data was recorded on either a TF30, TF30 Polara or Titan Krios microscope (FEI), equipped with K2 direct electron detectors and Quantum energy filters (Gatan). Tomographic data was collected with SerialEM (Mastronarde, 2005) or FEI Tomography, with pixel sizes between 2 and 3 Å/pixel at the specimen level and the energy selecting slit set to 20 eV, with a collection scheme of ±45° tilts (starting from 0° up, and returning to 0° to collect the negative tilts) with a 3° tilt interval. 7-10 image frames (0.2 s exposure/frame) in counting mode were collected per tilt at a dose rate of ~5e$^-$/unbinned pix/sec, giving an overall dose of ~ 60 e$^-$/Å^2. Frames were aligned and filtered for radiation damage using Unblur (Grant and Grigorieff, 2015). Tomograms were reconstructed using IMOD (Kremer et al., 1996). Contrast transfer functions were measured and data phase-flipped in IMOD. Tomograms were further processed for viewing in FIJI (Schindelin et al., 2012). Subvolumes were picked manually in IMOD and subvolume averaging and classification was done with PEET (Heumann et al., 2011). Subvolume averages and atomic models were visualised with USCF Chimera (Pettersen et al., 2004).

QUANTIFICATION AND STATISTICAL ANALYSIS

Subvolume averaging and classification was done using principal component analysis and k-means clustering as implemented in PEET. The number of subvolumes in the classes of undetermined RNCs were used as uncertainty ranges in the RNC binding measurements.

DATA AND SOFTWARE AVAILABILITY

The density maps corresponding to the average and 6 classes of RNCs bound to the native YidC membranes have been deposited in the Electron Microscopy Data Bank (EMDB) under ID codes 3909, 3919, 3920, 3921, 3922, 3923, and 3924.

Structure, Volume *26*

Supplemental Information

Combined ^{1}H-Detected Solid-State NMR Spectroscopy and Electron Cryotomography to Study Membrane Proteins across Resolutions in Native Environments

Lindsay A. Baker, Tessa Sinnige, Pascale Schellenberger, Jeanine de Keyzer, C. Alistair Siebert, Arnold J.M. Driessen, Marc Baldus, and Kay Grünewald

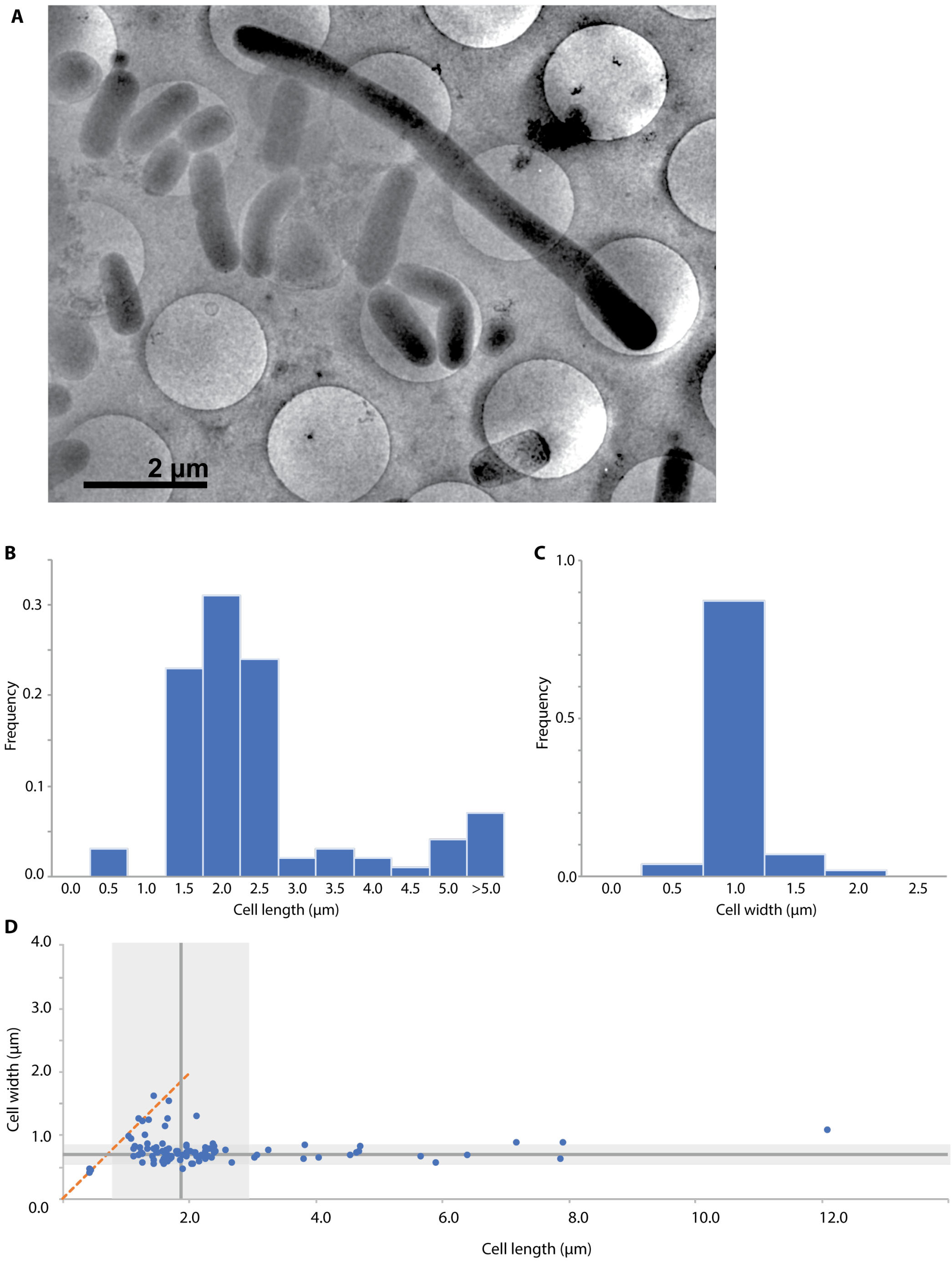

Supplemental Figure 1. (Related to Figure 2) The effect of rifampicin treatment on *E. coli* BL21 morphology. **A**) CryoEM projection image of *E. coli* BL21 expressing YidC after 15 hr rifampicin treatment at 25°C. Quantification of average cell length (**B**) and cell width (**C**) after rifampicin treatment. Most cells were between 1.0-2.5 µm long and 0.5-1.0 µm wide, but some were longer (<20%). **D**) Comparison of cell length versus cell width. Most cells maintained their rod shape, but a few exhibited a spherical morphology (orange dotted line). The median cell length was 1.86 µm (vertical grey line) with a standard deviation of the mean of 1.06 µm (shaded area either side). Typical cell lengths for *E. coli* when grown in glucose are 1.7-2.6 µm (Taheri-Araghi et al., 2015). The median cell width was 0.71 µm (horizontal grey line) with a standard deviation of the mean of 0.14 µm (shaded area either side).

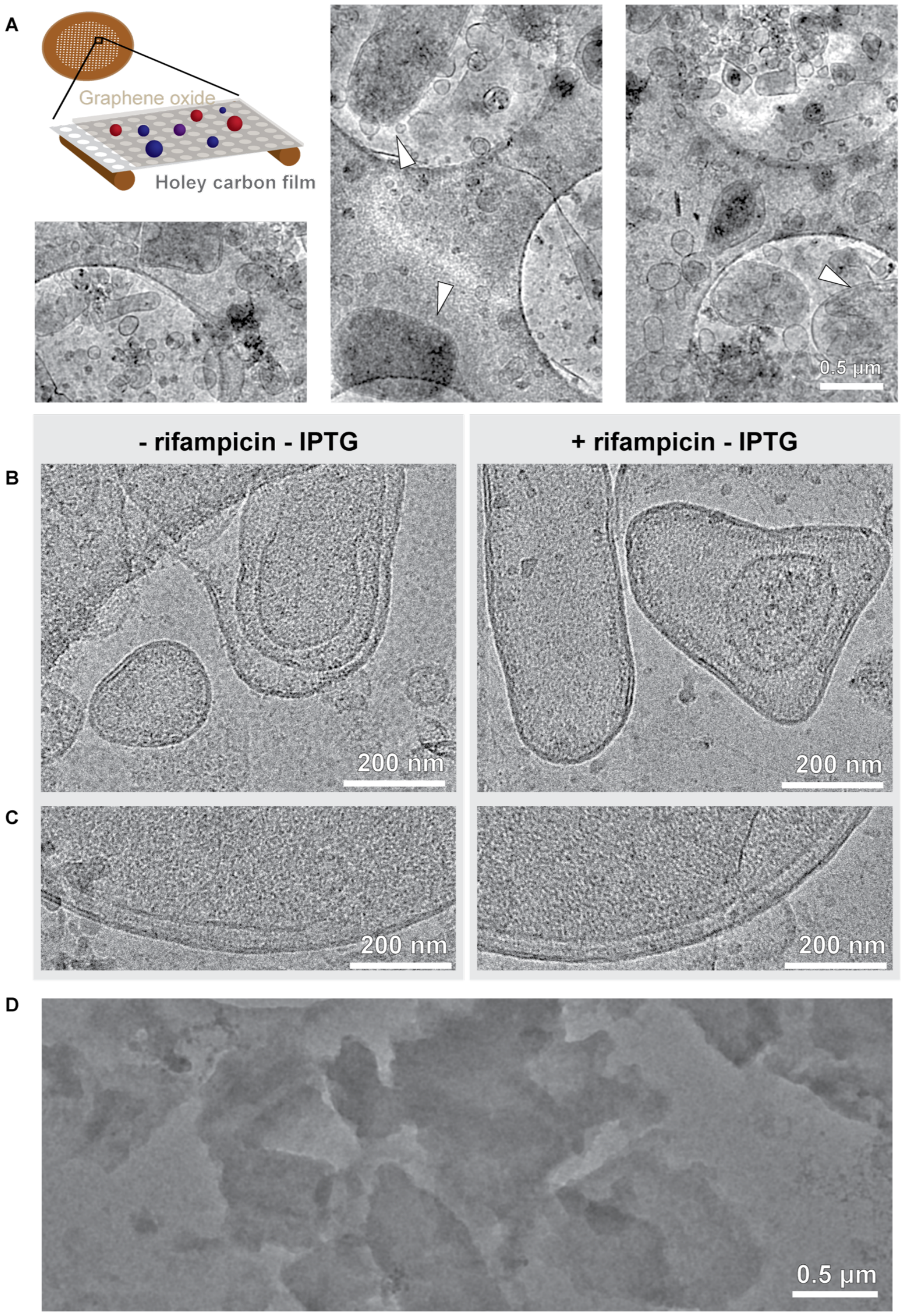

Supplemental Figure 2. (Related to Figure 2) Comparison of the morphology of cell envelope preparations with and without rifampicin and YidC over-expression. **A**) Cell envelope samples after rifampicin treatment and heterologous protein expression on graphene-oxide coated holey carbon grids (schematic, inset) display a variety of morphologies, including those that maintain the native cell pole (white arrows). **B**) No significant differences in the size or shapes of membranes was seen with or without rifampicin or YidC over-expression. **C**) No differences were observed in cell wall formation with or without rifampicin and YidC over-expression. **D**) Manipulation of cell envelope samples by standard methods used with synthetic bilayers (such as extrusion, freeze-thaws or sonication) produced a sheet morphology which is unsuitable for cryoET.

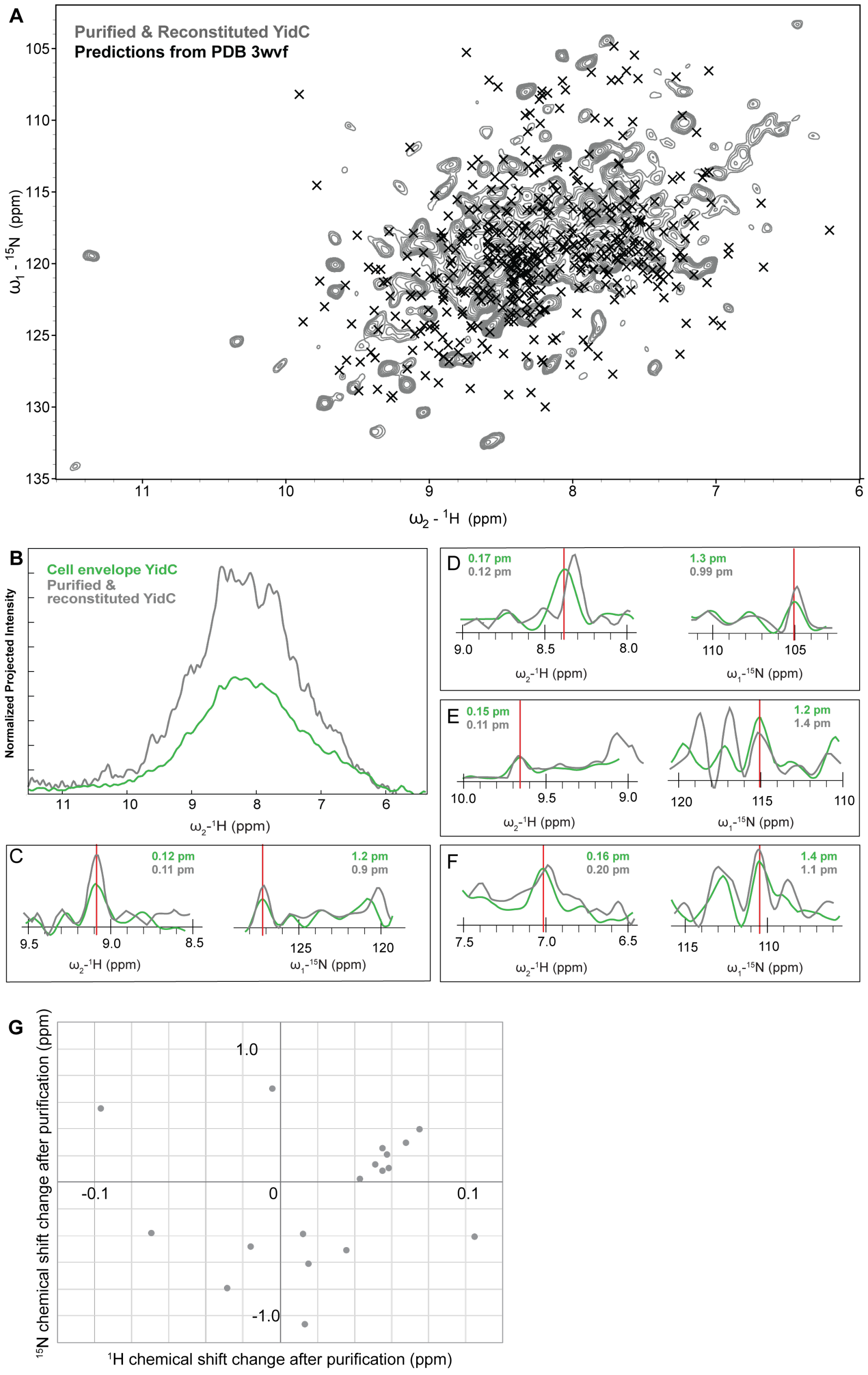

Supplementary Figure 3. (Related to Figure 3) **A)** Predictions of ssNMR resonances (black crosses) from Shiftx2 (Han et al., 2011) using the crystal structure of YidC (Kumazaki et al., 2014) (PDB 3wvf) do not agree well enough with our observed ssNMR spectrum (grey – purified and reconstituted YidC as in Figure 3) to assign resonances. **B)** Projection in the ^{15}N dimension of the 2D spectra from figure 3, normalized according to the square root of the number of scans used to acquire each spectrum. The concentration of YidC in the cell envelope samples is ~ 2x less than in the purified & reconstituted sample. **C-F)** Pairs of slices through the spectra in Figure 3 (cell envelope YidC – green; purified and reconstituted YidC – grey) for isolated peaks in the ^{1}H dimension (left) and ^{15}N dimension (right). The vertical red line in each spectrum indicates the position of the slice in the other dimension. The full width at half maximum intensity of the peak closest to red line is given in a top corner for both samples. Overall, line widths are often similar when comparing the two samples. **G)** Scatter plot showing the change in chemical shift for resolved peaks (assuming that the closest nearby correlation represents the same protein residue) in the purified and reconstituted spectrum in Figure 3, relative to the cell envelope spectrum.

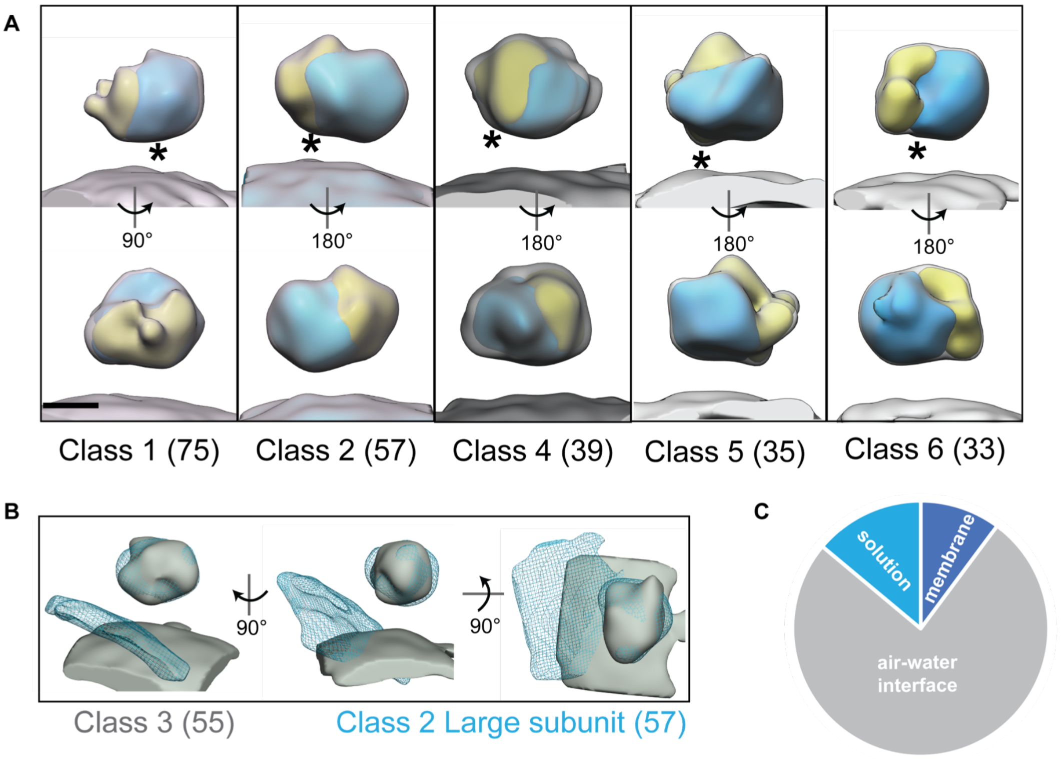

Supplementary Figure 4. (Related to Figure 5) Sub-tomogram averaging of YidC cell envelope-associated RNCs with Peet (Heumann et al., 2011). After aligning to an membrane-oriented average of all and then classifying with principle component analysis (PCA), particles were aligned to the class with the RNC in a YidC binding-competent conformation and then further classified by PCA. **A**) Five of the six classes (grey) with sufficient numbers for averaging were in YidC binding-competent positions, as judged by orientations of the large (blue) and small (yellow) ribosomal subunits (segmented manually) relative to the membrane. The approximate location where nascent chain would emerge from the exit channel is marked with an asterisk (*). Scale bar 10 nm. **B**) One class (grey) only had density for the large ribosomal subunit, which was in a different orientation relative to the membrane than those in the other classes (class 2 large subunit and membrane shown as blue mesh). **C**) Only ~25% of observed RNCs were available for analysis, due to the large number interacting with the air-water interface. As the system had time to reach equilibrium, this air-water interaction reduces the total concentration of RNCs available for binding but not the relative ratio of bound/unbound.
